# RIP1 inhibition protects retinal ganglion cells in glaucoma models of ocular injury

**DOI:** 10.1038/s41418-024-01390-7

**Published:** 2024-10-24

**Authors:** Bo Kyoung Kim, Tatiana Goncharov, Sébastien A. Archaimbault, Filip Roudnicky, Joshua D. Webster, Peter D. Westenskow, Domagoj Vucic

**Affiliations:** 1 Department of Ophthalmology Discovery, Pharmaceutical Research and Early Development, Roche Innovation Center Basel, F. Hoffmann-La Roche Ltd, Basel, Switzerland; 2 Institute of Chemical Sciences and Engineering (ISIC), École Polytechnique Fédérale de Lausanne (EPFL), Lausanne, Switzerland; 3 Department of Immunology Discovery, Genentech, 1 DNA Way, South San Francisco, CA USA; 4 Therapeutic Modalities, Pharmaceutical Research and Early Development, F. Hoffmann-La Roche Ltd, Basel, Switzerland; 5 Department of Pathology, Genentech, 1 DNA Way, South San Francisco, CA USA

**Keywords:** Immunochemistry, Cell death and immune response

## Abstract

Receptor-interacting protein 1 (RIP1, RIPK1) is a critical mediator of multiple signaling pathways that promote inflammatory responses and cell death. The kinase activity of RIP1 contributes to the pathogenesis of a number of inflammatory and neurodegenerative diseases. However, the role of RIP1 in retinopathies remains unclear. This study demonstrates that RIP1 inhibition protects retinal ganglion cells (RGCs) in preclinical glaucoma models. Genetic inactivation of RIP1 improves RGC survival and preserves retinal function in the preclinical glaucoma models of optic nerve crush (ONC) and ischemia–reperfusion injury (IRI). In addition, the involvement of necroptosis in ONC and IRI glaucoma models was examined by utilizing RIP1 kinase-dead (RIP1-KD), RIP3 knockout (RIP3-KO), and MLKL knockout (MLKL-KO) mice. The number of RGCs, retinal thickness, and visual acuity were rescued in RIP1-kinase-dead (RIP1-KD) mice in both models, while wild-type (WT) mice experienced significant retinal thinning, RGC loss, and vision impairment. RIP3-KO and MLKL-KO mice showed moderate protective effects in the IRI model and limited in the ONC model. Furthermore, we confirmed that a glaucoma causative mutation in optineurin, OPTN-E50K, sensitizes cells to RIP1-mediated inflammatory cell death. RIP1 inhibition reduces RGC death and axonal degeneration following IRI in mice expressing OPTN-WT and OPTN-E50K variant mice. We demonstrate that RIP1 inactivation suppressed microglial infiltration in the RGC layer following glaucomatous damage. Finally, this study highlights that human glaucomatous retinas exhibit elevated levels of *TNF* and *RIP3* mRNA and microglia infiltration, thus demonstrating the role of neuroinflammation in glaucoma pathogenesis. Altogether, these data indicate that RIP1 plays an important role in modulating neuroinflammation and that inhibiting RIP1 activity may provide a neuroprotective therapy for glaucoma.

## Introduction

Glaucoma is a chronic neurodegenerative disease and a leading cause of blindness that is projected to affect 112 million people worldwide by 2040 [1]. It is a complex multifactorial disease characterized by degeneration of retinal ganglion cells (RGC), which are projection neurons that relay visual cues to visual centers in the brain [2]. However, the pathophysiology underlying RGC death in glaucoma remains unclear. Although intraocular pressure (IOP) is a major modifiable risk factor for glaucoma progression, only a small proportion of patients with chronic ocular hypertension develops features of glaucoma [3, 4]. In fact, only around 10% of glaucoma patients present with elevated IOP above 22 mmHg [5]. Furthermore, meta-analysis studies report that the majority of glaucoma patients present with ocular pressures below 21 mmHg [6–8]. Thus, understanding IOP-independent mechanisms of glaucoma pathogenesis is crucial for developing medicines that can treat a broader range of glaucoma patients.

While neuroinflammation has been recognized as an important pathological factor in neurodegenerative diseases, tumor necrosis factor (TNF) has been suggested as a primary proinflammatory mediator in glaucoma [9–11]. Elevated levels of TNF in ocular biofluids (aqueous humor) and plasma are measurable in primary open-angle glaucoma (POAG) patients, the most prevalent type of glaucoma [12], compared to non-glaucomatous individuals [13–17]. In addition to TNF, TNF receptor 1 (TNFR1) is also increased in RGCs and in the optic nerve heads of glaucomatous eyes, where RGC axons exit the eye and further project to the brain [18–20].

Several genetic risk factors for glaucoma have been identified, including mutations in the *Optineurin* (*OPTN*) gene, which encodes a ubiquitin-binding protein OPTN [21]. The OPTN Glu50Lys (OPTN-E50K; rs28939688) missense mutation is linked with autosomal dominant forms of the disease [22], and patients harboring OPTN-E50K mutations are predisposed to severe forms of glaucoma and have poor prognoses [23]. Cellular studies have shown that OPTN-E50K induces cell death in an expression level-dependent manner [24]. Moreover, glaucomatous neurodegenerative features are observed in retinal organoids harboring OPTN-E50K mutations [25]. RGC loss is observed in OPTN-E50K knock-in mice, along with reactive gliosis [26, 27], progressive axonal degeneration, chronic inflammation, and visual impairments [27–29]. However, the mechanism by which OPTN-E50K contributes to RGC loss and axonal degeneration, ultimately leading to visual impairment, is not well understood. Earlier reports indicate that proinflammatory signatures of OPTN-deficient mice are regulated by TNF and receptor-interacting protein (RIP1, RIPK1)-mediated axonal degeneration [30, 31]. RIP1-dependent cell death can be triggered by TNF, toll-like receptors (TLR), or ischemic injury, leading to the activation of RIP1-mediated apoptosis and necroptosis [32, 33]. RIP1 is a promising therapeutic target for neurodegenerative diseases, including Alzheimer’s disease, amyotrophic lateral sclerosis (ALS), and multiple sclerosis, as well as acute neurological conditions such as stroke and traumatic brain injuries [34, 35].

In this study, we detected elevated TNF levels in glaucoma patients and demonstrated that OPTN-E50K operates via RIP1 to regulate RGC death. In glaucoma preclinical models, blocking RIP1 promoted RGC survival and preserved visual function by suppressing neuroinflammation in vitro and in vivo. Therefore, our data suggest that RIP1 inhibition may be a novel therapeutic option for glaucoma and potentially other neurodegenerative diseases implicated in neuroinflammation.

## Results

### RIP1 regulates RGC cell survival in mice with optic nerve crush (ONC) injuries by suppressing innate immune responses

**Fig. 1:**
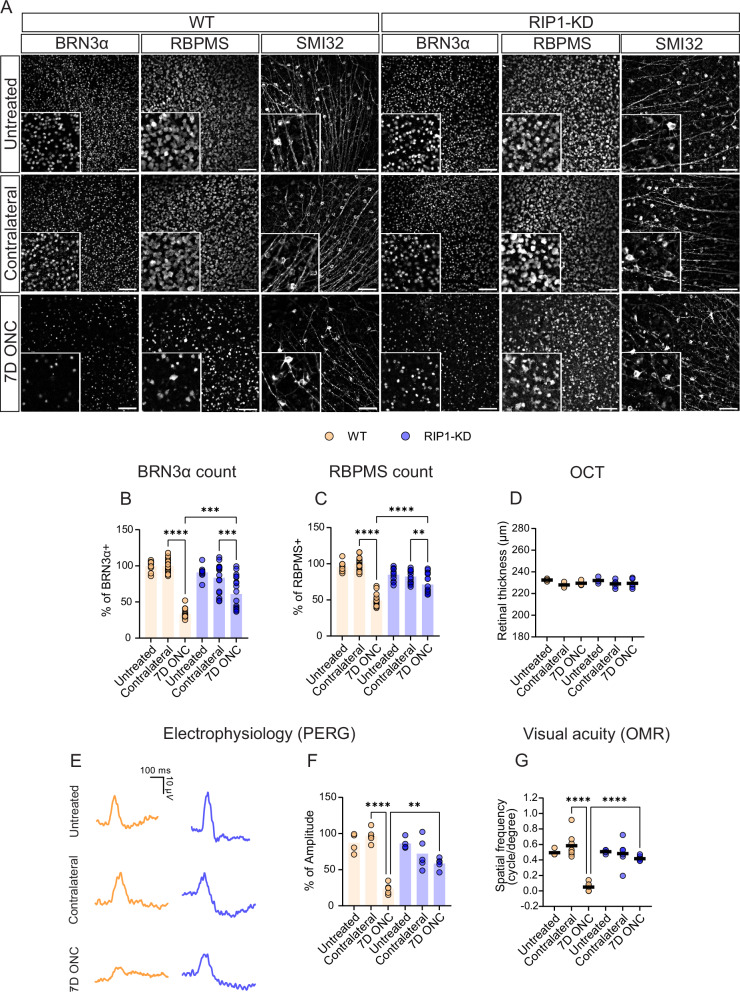
Genetic inactivation of RIP1 protects the RGC in an ONC glaucoma preclinical model. ONC was induced in WT and RIP1-KD mice and RGC survival was evaluated post-ONC. **A** Representative images from the retinal flat mount of each condition, including untreated (intact retina), contralateral (internal control), and at 7 days post-ONC. Scale bar = 100 μm. **B**, **C** Quantification of the number of RGCs. **B** BRN3α and **C** RBPMS-positive cells were counted as RGC. *n* = 8–10 mice per group. **D** Quantification of the thickness of the retina measured by optical coherent tomography (OCT) at 7 days post-ONC. *n* = 3–5 mice per group. **E** Representative graph of pattern electroretinogram (PERG) recording from each condition including untreated, contralateral, and 7 days post-ONC. **F** Quantification of PERG recording following ONC. *n* = 4–5 mice per group. **G** Visual acuity test with optomotor response (OMR) evaluation at 6 days post-ONC. *n* = 5–8 mice per group. Two-way ANOVA. **P < 0.01, ***P < 0.001, ****P < 0.0001.

To determine the role of RIP1 in glaucoma, RGC survival, and function were monitored in RIP1-kinase-dead (RIP1-KD, *Ripk1*^*D138N*^) mice with optic nerve crush (ONC) injury, which are used to model relevant features of retrograde glaucomatous stress (Supplementary Fig. 1A–C). Brain-specific homeobox/POU domain protein 3A (BRN3α) and RNA-binding protein with multiple splicing (RBPMS) were used as markers to quantify RGC survival. RIP1-KD mice showed a significantly higher number of BRN3α-positive cells (61.0%) and RBPMS-positive cells (71.1%) compared to WT mice (33.8% and 49.5%, respectively) 7 days post-ONC injury (Fig. 1A–C). The phenotype of axonal degeneration visualized by non-phosphorylated neurofilament H (SMI32) was more prominent in WT mice with ONC injury than RIP1-KD mice (Fig. 1A). In vivo imaging with optical coherence tomography (OCT) showed that retinal thickness remained unchanged in WT and RIP1-KD ONC mice (Fig. 1D). In addition, RGC functional activity monitored by pattern electroretinogram (PERG) was rescued in RIP1-KD mice (58.6%) compared to WT mice (18.0%) (Fig. 1E, F). Visual acuity measured by optomotor responses (OMR) was also rescued in RIP1-KD mice (0.389 cycles per degree (RIP1-KD) vs. 0.056 cycles per degree (WT; Fig. 1G).

**Fig. 2:**
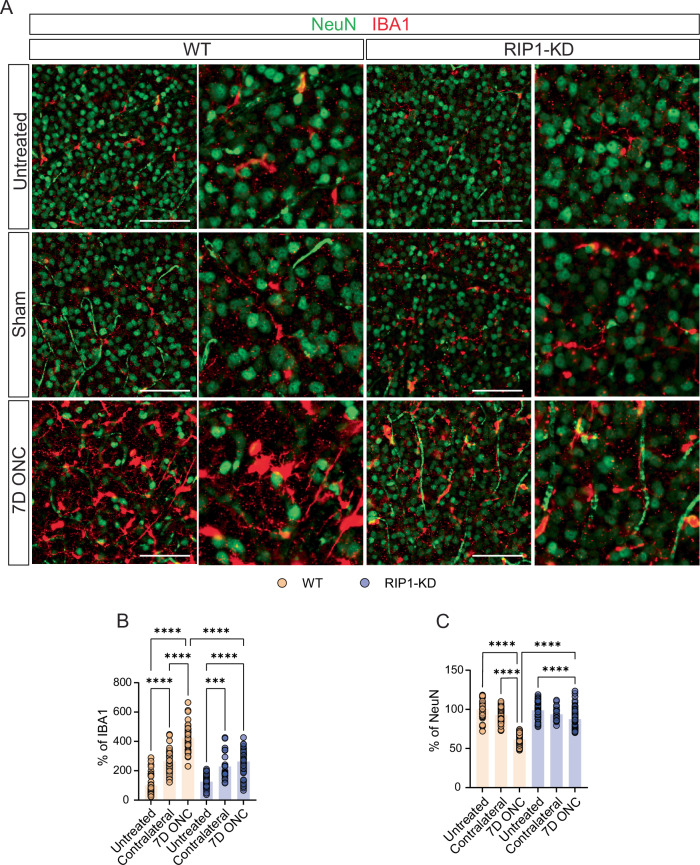
Genetic inactivation of RIP1 suppresses infiltrating microglia in the RGC layer following ONC damage. **A** Representative images of RGC layer (GCL) from an ONC glaucoma model at 7 days post-ONC. Quantification of **B** IBA1 and **C** NeuN-positive cells in WT and RIP1-KD mice after ONC. *n* = 5–8 mice per group. Scale bar = 100 μm. Two-way ANOVA. ***P <  0.001, ****P < 0.0001.

In response to injury or inflammation, activated microglia can damage or phagocytose neurons, resulting in neuronal loss and dysfunction [36]. Therefore, the status of microglia under glaucomatous-like stress in both WT and RIP1-KD mice was investigated by counting the number of ionized calcium-binding adaptor molecule 1 (IBA1)-positive cells in RGC layers. Microglial infiltration to the RGC layer and decreased numbers of neuronal nuclear protein (NeuN)-positive cells were noted in WT mice after ONC (Fig. 2A–C). In contrast to WT, RIP1-KD mice showed less microglia infiltration and a higher number of NeuN-positive cells following ONC (Fig. 2A–C). Collectively, these data suggest that RIP1 kinase activity contributes to RGC cell death in ONC-induced retrograde glaucomatous damage and underscores its role in retinal neuroinflammation.

The involvement of necroptosis pathways mediated by RIP1 in ONC was further examined by investigating the roles of RIP3 knockout (RIP3-KO) and MLKL knockout (MLKL-KO) mice in the ONC model. ONC in RIP3-KO mice resulted in reduced microglial infiltration, whereas MLKL-KO mice did not exhibit a decrease in microglia in the RGC layer (Supplementary Fig. S1D, E). The number of NeuN-positive cells indicated that RGCs were preserved post-ONC injury in both RIP3-KO and MLKL-KO mice compared to ONC in WT mice (Supplementary Fig. S1D, F). Surviving RGCs in RIP3-KO and MLKL-KO mice that underwent ONC showed a trend of improved PERG function by +25% and +19.1%, respectively, although these improvements were not statistically significant (Supplementary Fig. S1G, H). PERG data from ONC models in RIP3-KO and MLKL-KO mice were further translated to OMR data, as they did not show significant improvement in visual acuity (Supplementary Fig. S1I). Similar to the OCT evaluation of WT and RIP1-KD mice with ONC, ONC in RIP3-KO and MLKL-KO did not result in retinal thinning (Supplementary Fig. S1J). In summary, RIP1-KD mice exhibited the most significant protection effect in the ONC model, while ONC in RIP3-KO and MLKL-KO mice showed moderate/limited improvement (Supplementary Fig. S1K).

### RIP1 regulates RGC survival in a retinal ischemia–reperfusion injury (IRI) by suppressing innate immune responses

**Fig. 3:**
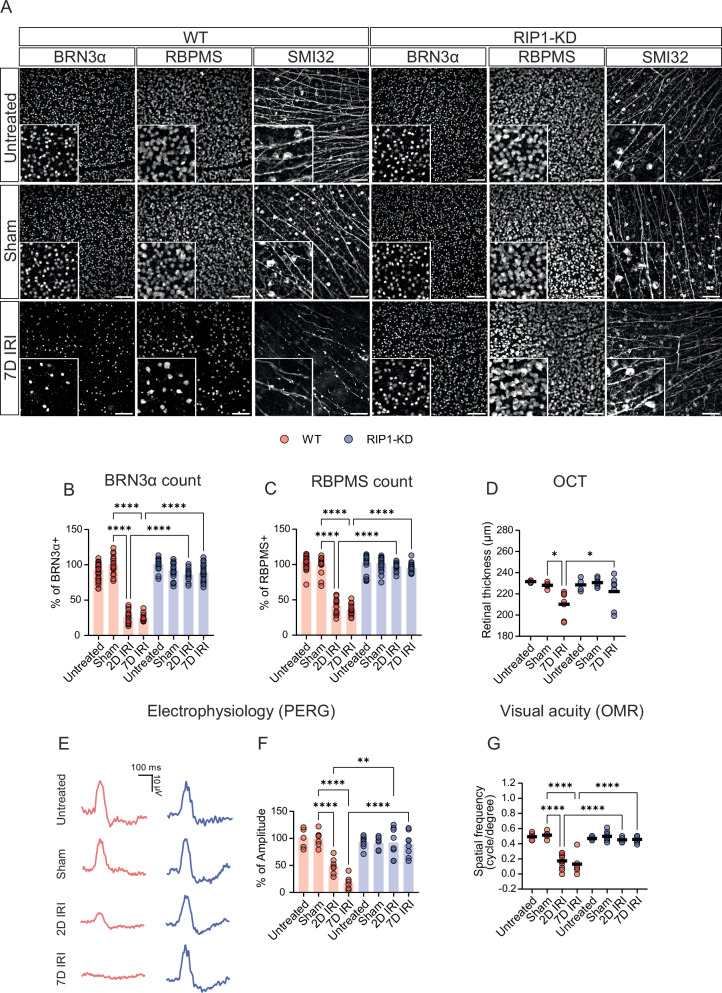
Genetic inactivation of RIP1 protects the RGC in an IRI glaucoma preclinical model. IRI was induced in WT and RIP1-KD mice and RGC survival was evaluated post-IRI. **A** Representative images from the retinal flat mount of each condition, including untreated (intact retina), sham, and at 7 days post-IRI. Scale bar  = 100 µm. **B**, **C** Quantification of the number of RGCs. **B** BRN3α and **C** RBPMS-positive cells were counted as RGCs. *n* = 6–8 mice per group. **D** Quantification of the thickness of the retina measured by optical coherence tomography (OCT) at 7 days post-IRI. *n* = 4–8 mice per group. **E** Representative graph of pattern electroretinogram (PERG) recording from each condition, including untreated (intact retina), sham, 2, and 7 days post-IRI. **F** Quantification of PERG recording following IRI. *n* = 5–8 mice per group. **G** Visual acuity test with optomotor response (OMR) evaluation at 6 days post-IRI. *n* = 5–8 mice per group. Two-way ANOVA. *P < 0.05, **P < 0.01, ****P < 0.0001.

To explore anterograde glaucomatous stress, the ischemia–reperfusion injury model (IRI) was induced in WT and RIP1-KD mice and RGC survival was examined (Supplementary Figs. S1C and S2A). Immunofluorescence analyses on 7 days post-IRI mouse retinas revealed that RIP1-KD mice exhibited more BRN3α (88.7%) and RBPMS (95.4%) positive cells compared to WT mice (24.5% and 35.5%, respectively) (Fig. 3A–C and Supplementary Fig. S2B). SMI32 visualized the structure of RGC axons and showed decreased number of cell bodies and thinner axon branches in WT than RIP1-KD mice at 7 days post-IRI (Fig. 3A). After IRI, retinal thickness values in RIP1-KD mice remained unchanged in contrast to WT mice, which exhibited reduced retinal thickness values, suggesting significant cell loss (Fig. 3D). At 2 and 7 days post-IRI, PERG values were significantly compromised in WT mice (48.8% and 18.4%, respectively), whereas the drop in PERG values was restored in RIP1-KD mice undergoing IRI (92.4% and 89.1% versus sham control, respectively; Fig. 3E, F). Moreover, RIP1-KD mice preserved visual acuity measured by OMR following IRI (0.444 cycles per degree both at 2 and 7 days post-IRI), whereas visual acuity in WT mice with IRI was significantly compromised (0.1667 and 0.111 cycles per degree at 2 and 7 days post-IRI, respectively) (Fig. 3G).

**Fig. 4:**
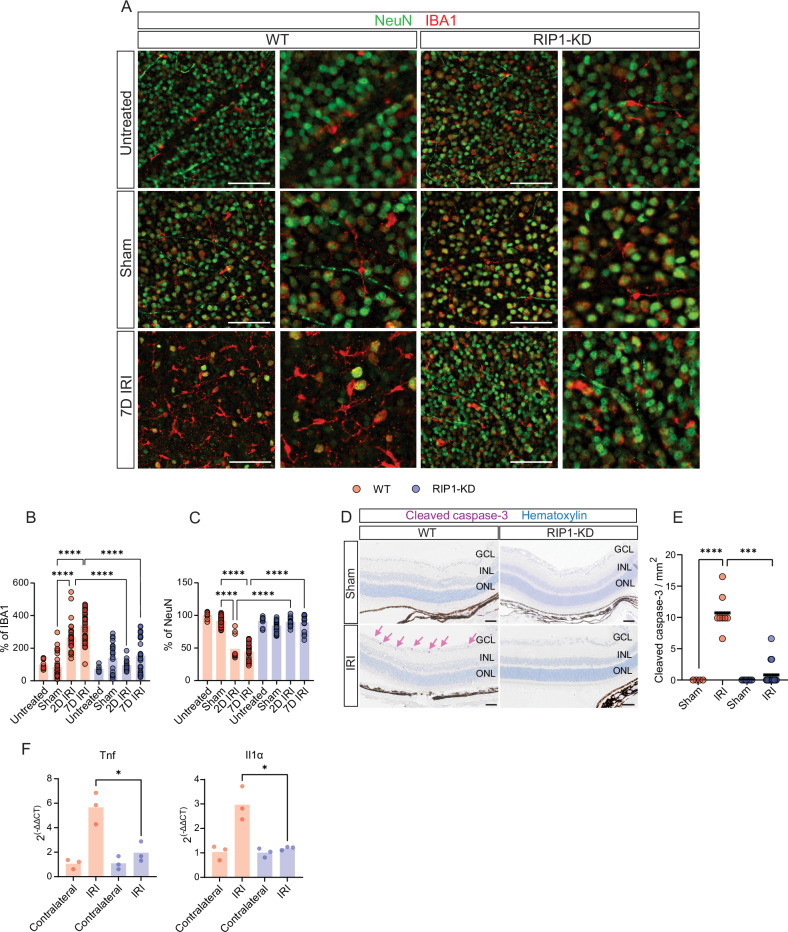
Genetic inactivation of RIP1 suppresses infiltrating microglia in the RGC layer following IRI damage. **A** Representative images of the RGC layer (GCL) from IRI-induced glaucoma model at 7 days post-IRI and **B** quantification of IBA1 and **C** NeuN-positive cells in both WT and RIP1-KD mice. *n* = 4–8 mice per group. Scale bar = 100 µm. **D** Representative images of cleaved caspase-3 immunolabeling. The retinas from WT and RIP1-KD mice underwent IRI. **E** Quantification of cleaved caspase-3 positive cells. **F** The level of *TNF* and *IL-1α* transcripts in WT and RIP1-KD mice eyes with IRI were examined using qPCR. GCL ganglion cell layer, INL inner nuclear layer, ONL outer nuclear layer. Scale bar = 50 µm. Two-way ANOVA. *P < 0.05, ***P < 0.001, ****P < 0.0001.

Similar to observations in the ONC model, at 7 days post-IRI, the number of infiltrating microglia in retinas from RIP1-KD mice did not change, but the number was increased in WT IRI mice (Fig. 4A, B). Also, fewer NeuN-positive cells were detectable in the RGC layer of WT IRI mice (Fig. 4C). In support of IRI-induced cell death, cleaved caspase-3 was detected in the RGC layer and was elevated in WT IRI mice while RIP1-KD mice showed less cleaved caspase-3 signals (Fig. 4D, E). Thus, genetic inactivation of RIP1 protects RGCs in IRI-induced anterograde glaucomatous damage and suppresses aberrant innate immune activation. This immune activation was further confirmed by the upregulated levels of *Tnf* and *Il-1α* expression in IRI in WT mice compared to RIP1-KD mice (Fig. 4F). Based on these results, the pharmacological inhibition of RIP1 was tested to see if it could prevent tissue damage in IRI mice. BRN3α and RBPMS-positive RGCs were significantly protected in mice treated with the RIP1 inhibitor, GNE684, while these RGCs were decreased in the WT mice retinas after IRI (Supplementary Fig. S2C–E).

To investigate the involvement of RIP3 and MLKL in IRI-related glaucoma pathogenesis, RIP3-KO and MLKL-KO mice were subjected to IRI. IRI in RIP3-KO and MLKL-KO mice resulted in significantly reduced microglial infiltration in RGC layers (Supplementary Fig. S3A, B). RGCs visualized by NeuN labeling demonstrated RGC survival post-IRI in both RIP3-KO and MLKL-KO mice compared to WT mice (Supplementary Fig. S3A, C). Persistent RGCs in RIP3-KO mice following IRI resulted in a significant improvement in PERG values by +39.1% as well as in visual acuity, as validated by OMR measurements. MLKL-KO mice also showed an improvement in visual acuity after IRI, although the +16% improvement in PERG value was not statistically significant (Supplementary Fig. S3D–F). OCT images also demonstrated that RIP3-KO and MLKL-KO mice with IRI preserved the thickness of the retina (Supplementary Fig. S3G). In summary, RIP1 genetic inactivation, or RIP3 and MLKL ablation largely inhibited retinal degeneration induced by IRI (Supplementary Fig. S3H).

### OPTN deletion and E50K mutations sensitize retinal neurons to TNF/RIP1-mediated necroptosis

**Fig. 5:**
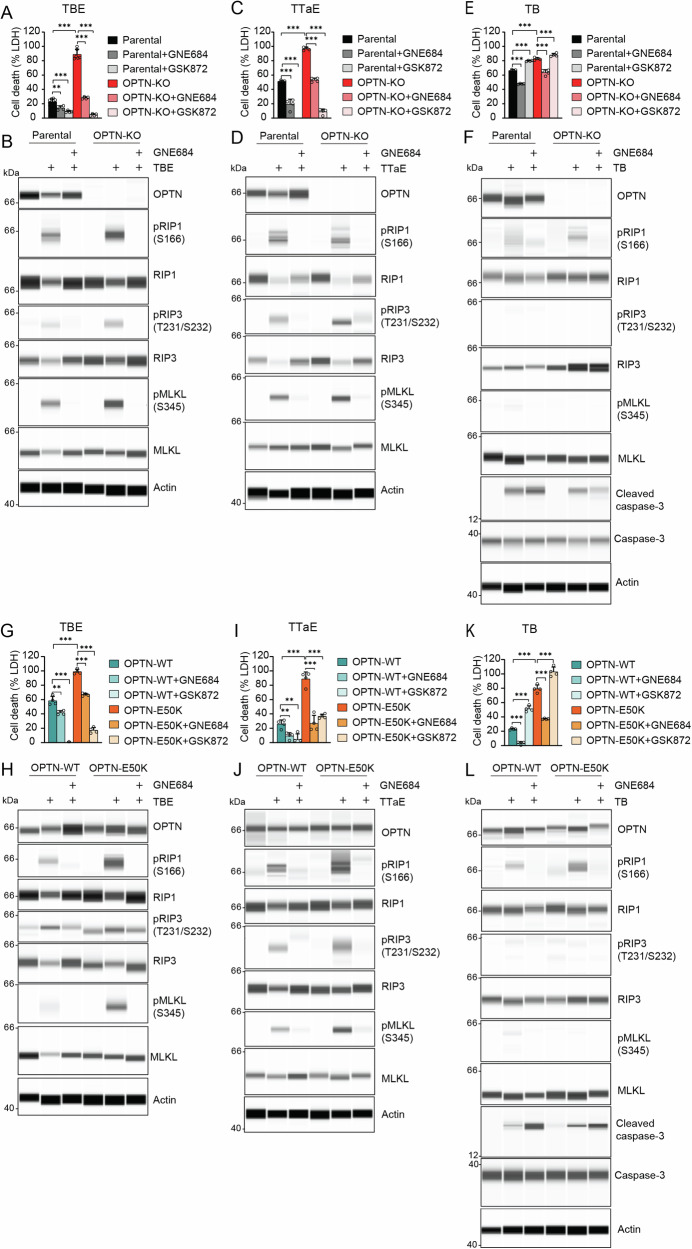
OPTN deletion and OPTN-E50K mutation sensitize cells to RIP1-mediated inflammatory cell death. RIP1-dependent cell death was induced in 661w parental OPTN-KO, OPTN-WT, and OPTN-E50K cells. Cell death was induced by TNF, BV6, and Emricasan (TBE; **A**, **B**, **G**, **H**), TNF, TAK1 inhibitor, and Emricasan (TTaE; **C**, **D**, **I**, **J**), or TNF and BV6 (TB; **E**, **F**, **K**, **L**). **A** 661w parental and OPTN-KO cells were treated with TBE (100 ng mL^−1^ TNF, 2.5 μM BV6, and 10 μM Emricasan) and 20 μM GNE684 or 10 μM GSK872. LDH assay. **B** Immunoblot assay for indicated proteins in 661w parental and OPTN-KO cells after 4 h treatment with TBE and GNE684. **C** 661w parental and OPTN-KO cells were treated with TTaE (100 ng mL^−1^ TNF, 0.6 μM Takinib, and 10 μM Emricasan) and 20 μM GNE684 or 10 μM GSK872. LDH assay. **D** Immunoblot assay for indicated protein in 661w and OPTN-KO cells after 4 h treatment with TTaE and GNE684. **E** 661w parental and OPTN-KO cells were treated with TB (100 ng mL^−1^ TNF and 2.5 μM BV6) and 20 μM GNE684 or 10 μM GSK872. LDH assay. **F** Immunoblot assay for indicated protein in 661w and OPTN cells after 4 h treatment with TB and GNE684. **G** OPTN-WT and OPTN-E50K cells were treated with TBE and GNE684 or GSK872. LDH assay. **H** Immunoblot assay for indicated protein in OPTN-WT and OPTN-E50K cells after 4 h treatment with TTaE and GNE684. **I** OPTN-WT and OPTN-E50K cells were treated with TTaE and GNE684 or GSK872. LDH assay. **J** Immunoblot assay for indicated protein in OPTN-WT and OPTN-E50K cells after 4 h treatment with TTaE and GNE684. **K** OPTN-WT and OPTN-E50K cells were treated with TB and GNE684 or GSK872. LDH assay. **L** Immunoblot assay for indicated protein in OPTN-WT and OPTN-E50K cells after 4 h treatment with TB and GNE684. Two-tailed Student *t* test. Data in columns represent mean ± SD. *P < 0.05, **P < 0.01, ***P < 0.001.

Having demonstrated that blocking RIP1 activity provides benefits in two different ocular glaucoma models, this study explored whether known glaucoma risk factors predispose to RIP1-mediated cell death and tissue injury. E50K mutation in OPTN is associated with severe forms of glaucoma and a worse prognosis [22]. OPTN is also an important adaptor for TANK-binding kinase 1 (TBK1), a kinase that is known to suppress the activation of RIP1 [37]. To study the function of OPTN and its pathogenic variant OPTN-E50K, OPTN-knockout (OPTN-KO), OPTN-WT, and OPTN-E50K cell lines were generated in murine neuroretinal 661w cells, which express critical components of the TNF/RIP1-mediated cell death signaling pathway (Supplementary Fig. S4A–E). To generate OPTN-KO cells, combinations of two different sgRNAs were introduced into 661w cells (Supplementary Fig. S4A, B). The engineered OPTN-KO cells were more sensitive to the necroptotic stimulus (TBE; TNF, IAP antagonist BV6, and caspase inhibitor Emricasan) compared to the parental 661w cell line (Supplementary Fig. S4C). Next, we genetically engineered OPTN-KO cells to reconstitute either the WT variant of OPTN (OPTN-WT) or the pathogenic variant OPTN-E50K (Supplementary Fig. S4D, E). The 661w parental, OPTN-KO, OPTN-WT, and OPTN-E50K cells were treated with TBE to investigate the contribution of OPTN to TNF-mediated necroptosis. TBE treatment induced more death in OPTN-KO cells than parental cells, and co-treatment with RIP1 (GNE684) or RIP3 (GSK872) inhibitors prevented cell death (Fig. 5A). In addition, higher phosphorylation of RIP1, RIP3, and MLKL was observed in OPTN-KO cells with TBE treatment and was suppressed by RIP1 inhibition (Fig. 5B). Another necroptosis-inducing combination, TTaE (TNF, TAK1 inhibitor Takinib and Emricasan) caused higher levels of cell death in OPTN-KO compared to parental cell lines and enhanced phosphorylation of RIP1, RIP3, and MLKL proteins, while GNE684 suppressed this cell death (Fig. 5C, D). The role of OPTN in RIP1-mediated apoptosis was next investigated with TNF plus BV6 treatment, which increased cell death in OPTN-KO cells compared to parental cells (Fig. 5E), and GNE684 effectively suppressed TB-induced cell death and RIP1 phosphorylation (Fig. 5F).

Similar to OPTN-KO cells, OPTN-E50K cells exhibited increased cell death and phosphorylation of RIP1, RIP3, and MLKL following TBE treatment (Fig. 5G, H). Our observation that OPTN-E50K cells were more sensitive to RIP1-dependent cell death was further confirmed using PiggyBac gene delivery technology to reconstitute OPTN expression (Supplementary Fig. S4F). Similar to TBE treatment, TTaE-induced cell death was more prominent in the OPTN-E50K line compared to the OPTN-WT cells (Fig. 5I). Higher phosphorylation of RIP1, RIP3, and MLKL detected in OPTN-E50K cells was prevented by GNE684 treatment (Fig. 5J). In addition, TB treatment induced more cell death in OPTN-E50K cells compared to the OPTN-WT line (Fig. 5K, L).

As expected, RIP1-independent apoptosis inducers, including doxorubicin, staurosporine, and a combination of TNF and cycloheximide, did not result in phosphorylation of RIP1, RIP3, and MLKL, but the cleaved PARP levels were elevated (Supplementary Fig. S4G–I). Furthermore, RIP1-independent apoptosis inducers did not further enhance cell death in OPTN-KO and OPTN-E50K cell lines (Supplementary Fig. S4J–O). Collectively, our results suggest that the E50K mutation sensitizes 661w cells to TNF-induced and RIP1-dependent cell death, similar to the response observed in OPTN-KO cells.

### OPTN-E50K mediated RGC death is ameliorated by RIP1 inhibition in ocular IRI mice

**Fig. 6:**
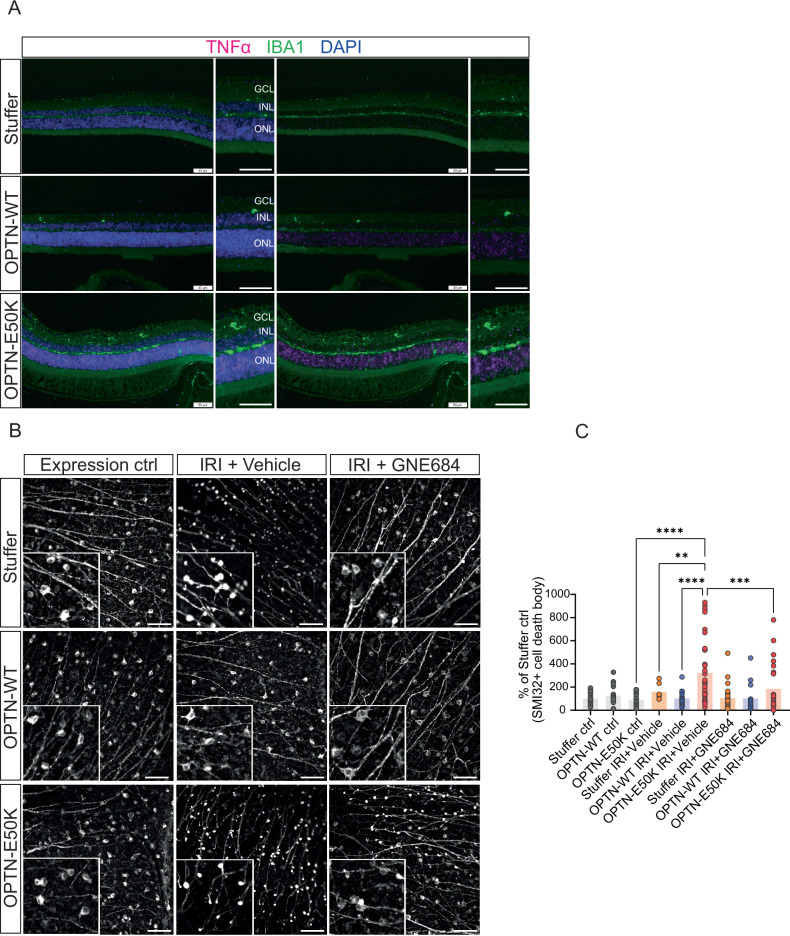
Pharmacological RIP1 inhibition attenuates IRI damage in RGC axons. **A** AAV2s carrying stuffer, OPTN-WT, and OPTN-E50K sequences were injected into the retinas. AAV2 transduced retinas were co-labeled with IBA1 immunolabeling and in situ hybridization of TNF. Scale bar = 50 μm. **B** Representative images from retinal whole-mount immunohistochemistry of SMI32. Retinas were transduced with stuffer, OPTN-WT, or OPTN-E50K and underwent IRI. AAV2 transduced mice were administrated with GNE684 100 mg kg^−1^ by oral gavage, twice per day throughout IRI. **C** Quantification of SMI32-positive shrunken cells. *n* = 3–4 mice per group. Scale bar = 100 µm. Data represented in a bar graph. Two-way ANOVA. **P < 0.01, ***P < 0.001, ****P < 0.0001.

Our data suggest that OPTN-E50K sensitizes cells to RIP1-dependent inflammatory cell death. To further elucidate the role of RIP1 and OPTN-E50K in RGC cell death in vivo, OPTN-WT and OPTN-E50K transgenes were induced using adeno-associated virus 2 (AAV2) and intravitreal injections. Firstly, the successful transduction of RGCs and their axons by AAV2 was confirmed using optical coherent tomography-angiogram (OCT-A) in vivo imaging and confocal ex vivo imaging (Supplementary Fig. S5A–C). In addition, an immunoblotting assay confirmed that transgenes including OPTN-WT and OPTN-E50K expressed OPTN (Supplementary Fig. S5D). Transgenic expression of OPTN-WT and OPTN-E50K delivered by AAV2 promoted RIP1 activation, and OPTN-E50K transduced cells treated with TBE showed higher RIP1 phosphorylation compared to OPTN-WT transduced retinal cells (Supplementary Fig. S5E). In vivo, elevated *TNF* mRNA and increased numbers of activated microglia (IBA1+ cells) were observed in AAV2-OPTN-E50K transduced retinas compared to OPTN-WT and stuffer control AAV2 transduced retinas (Fig. 6A). RGC axons, visualized by SMI32 staining, were not affected by empty AAV2 (Fig. 6B). Mice transduced with AAV2-stuffer controls, OPTN-WT, or OPTN-E50K were subjected to IRI. After IRI, SMI32-positive cell bodies in the stuffer control and OPTN-E50K transduced retinas appeared shrunken and condensed compared to OPTN-WT transduced retinas (Fig. 6B, C). Cell shrinkage is a known characteristic of cell death as well as a sign of glaucomatous phenotype [38], so the shrunken SMI32-positive cell bodies were counted as dead. The number of SMI32-positive dead cell bodies remained unchanged in untreated retinas (Fig. 6C). However, OPTN-E50K transduced retinas showed higher numbers of SMI32-positive dead cell bodies compared to OPTN-WT and stuffer control transduced retinas after IRI, but this was reduced by GNE684 administration (Fig. 6B, C). In conclusion, SMI32 immunostaining revealed a significantly higher number of dead cell bodies in vulnerable RGC axons of OPTN-E50K transduced retinas during IRI in a RIP1 kinase-dependent fashion, emphasizing the potential of RIP1 inhibition to limit axonal degeneration after IRI-induced anterograde glaucomatous stress.

### Proinflammatory mediators are detectable in glaucoma patient retinas

**Fig. 7:**
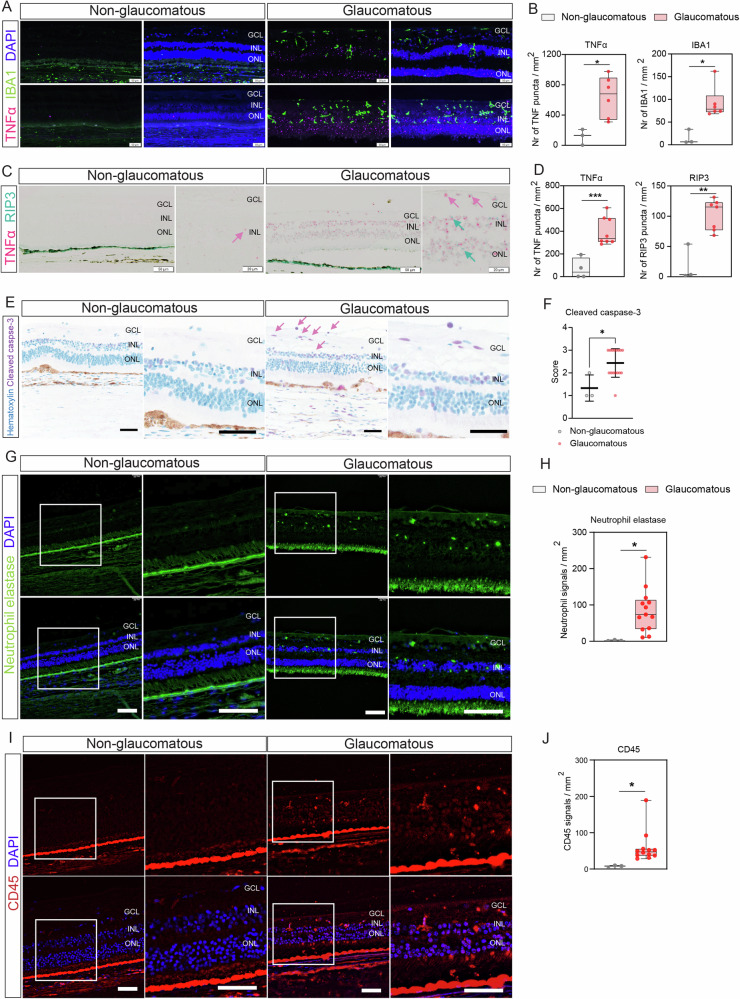
Human glaucomatous retinas exhibit proinflammatory cell death phenotypes. Representative images of human non-glaucomatous (*n* = 3 eyes) and glaucomatous (*n* = 6 eyes) retinas were included in the study (Supplementary Table 1 and Supplementary Fig. 7). Glaucomatous retinas were collected from patients who were clinically diagnosed with glaucoma before death. **A** Representative images of postmortem glaucomatous (ID: 140099 and 150001) and non-glaucomatous human retinas (ID: 140001 and 140016) were co-labeled with IBA1 immunolabeling and in situ hybridization (ISH) of *TNF* and **B** *TNF* ISH quantification. Scale bar = 50 μm. **C** Dual ISH representative images for *TNF* (pink) and *RIP3* (green) from postmortem human glaucomatous (ID: 140149) and non-glaucomatous retinas (ID: 140001) and **D** its quantification. 20x Olympus scanner microscope. Scale bar = 20 μm and 50 μm. The arrows indicate the *TNF* (pink) and *RIP3* (green) signals from the retina tissue. **E** Representative images from postmortem glaucomatous human retina. Immunolabeling of cleaved caspase-3 and **F** cleaved caspase-3 quantification. The arrows indicate the cleaved caspase-3 (pink) signals from the retina tissue. Scale bar = 50 μm. **G** Representative immunohistochemistry images for Neutrophil elastase (green) staining from postmortem human glaucomatous (ID: 150001) and non-glaucomatous (ID: AE02120) retina and **H** quantification. 20x Leica Thunder microscope. Scale bar = 100 μm. **I** Representative immunohistochemistry images for CD45 (red) staining from postmortem human glaucomatous (ID: 150001) and non-glaucomatous (ID: 140015) retina and **J** quantification. ×20 Leica Thunder microscope Scale bar = 100 μm. GCL ganglion cell layer, INL inner nuclear layer, ONL outer nuclear layer. Two-tailed Student’s *t* test. *P < 0.05, **P < 0.01, ***P < 0.001.

It was previously reported that upregulated TNF and RIP3 levels in human glaucoma patient retinas are linked to neurodegeneration [39, 40]. Therefore, in situ hybridization was used to confirm *TNF* and *RIP3* upregulation in cross-sectioned patient retinal samples [40, 41] (Supplementary Table 1; Fig. 7A and Supplementary Figs. S6 and S7A, B). The tissues were also stained for IBA1, as IBA1-positive cells are one of the primary sources of TNF in the retina [42, 43]. Human glaucomatous retinas exhibited elevated levels of *TNF* and higher numbers of IBA1-positive cells compared to non-glaucomatous retinas (Fig. 7A, B and Supplementary Fig. S7A). In glaucoma eyes, IBA1-positive cells were detectable in the inner retinal layers, including the retinal ganglion cell layer (RGL), the inner nuclear layer (INL), and the outer nuclear layer (ONL) (Fig. 7A and Supplementary Fig. S7A). Furthermore, in situ hybridization revealed upregulation of *TNF* and *RIP3* in human glaucomatous retina tissues, particularly in the RGL, INL, and ONL (Fig. 7C, D and Supplementary Fig. S7B). In addition, increased levels of cleaved caspase-3 signal were detected in human glaucomatous tissues (Fig. 7E, F). To examine innate immune activation, human retinal slides were immunolabeled for neutrophil elastase and CD45 to visualize immune cell infiltration in the retina (Fig. 7G–J and Supplementary Fig. S7C, D). Neutrophils that were visualized by neutrophil elastase labeling showed a higher signal in the inner layer of the glaucomatous retina in comparison to the human retinas that do not have glaucoma history (Fig. 7G, H and Supplementary Fig. S7C). In addition, leukocytes that were visualized by CD45 also showed an elevated level of signals in the glaucomatous retina than non-glaucomatous human retina (Fig. 7I, J and Supplementary Fig. S7D). These findings highlight that inflammatory mediators are present in glaucoma eyes in a pattern consistent with RGC damage and cell death.

## Discussion

Early neuroinflammatory responses are triggered by astrocytes and microglia in the context of glaucoma [44]. Despite the ambiguous nature of the specific stimuli for inflammatory reactions in glaucoma, it is apparent that inflammatory processes, modulated in part by astrocytes and resident microglia, play a pivotal role in the pathogenesis of glaucoma [42]. Numerous studies with clinical and preclinical data have unveiled the early upregulation of genes associated with inflammatory pathways in both the retina and optic nerve head in glaucoma [10, 11, 45–48]. Cumulatively, these studies emphasize the upregulation of inflammatory phenotypes in human glaucoma.

In this study, a comprehensive evaluation of two experimental glaucoma models, ONC and IRI, was conducted in mice lacking core proteins in the necroptosis pathway (RIP3-KO and MLKL-KO) or expressing a kinase-dead form of RIP1 (RIP1-KD). This approach delineates the specific contributions of necroptosis signaling and differential impacts of RIP1, RIP3, and MLKL in ONC and IRI models, which mimic retrograde and anterograde glaucomatous stresses in the retina. Our findings pinpoint potential therapeutic targets for mitigating RGC loss in glaucoma, underscoring the pivotal role of RIP1 in RGC death and optic nerve damage, and offer novel perspectives for developing targeted glaucoma interventions.

Inhibiting RIP1 in ONC and IRI preclinical glaucoma models significantly prevents inflammation and promotes RGC survival and function, with a greater effect observed in IRI preclinical glaucoma models compared to the ONC model. The absence of RIP3 or MLKL protected RGC function against IRI-induced retinal damage but to a lesser degree in the ONC model, where RGC death is predominantly mediated by apoptosis [49–52]. On the other hand, the protective effect of RIP3 and MLKL ablation in IRI-induced ocular damage has been reported in other tissues following IRI [35, 53–56]. Further evidence for the role of necroptosis in retinal injury could come from a newly published pMLKL immunohistochemistry assay in future studies [57]. Overall, our results are consistent with studies that implicated MLKL and RIP3 in retinal IRI [58, 59].

Collectively, our data suggest that RIP1 may regulate RGC survival via OPTN in cell autonomous as well as intercellular manners by fine-tuning innate immune responses. In addition, this study indicates that targeting RIP1 in glaucoma could improve patient outcomes by preventing RGC axonal degeneration and cell death, and by normalizing the innate immune response to restore retinal homeostasis. In addition, we present data that OPTN-E50K mutations sensitize RGCs to RIP1-mediated inflammation. Our findings substantiate that OPTN-E50K overexpression within the mouse retina led to heightened TNF levels, underscoring a proinflammatory trait, and concurrently, the inflammatory phenotype attributed to OPTN-E50K exacerbates cellular susceptibility to RIP1-dependent cell death. Prior investigations involving OPTN-E50K in transgenic mice and retinal organoids have consistently underscored its role in contributing to inflammation, notably marked by reactive gliosis and microglial activation [26, 29]. This finding further supports RIP1-mediated inflammation in glaucoma, as OPTN-E50K represents a monogenic form of glaucoma with causative pathogenic variants, indicating a direct relationship between these variants and the development of the disease [22, 23].

The protective effect of inhibiting RIP1 is particularly pronounced in the retinal IRI model. Necroptosis predominates over apoptosis in IRI-mediated cell death [60, 61]. Besides being implicated in the eyes as outlined in this study, previous articles emphasize that RIP1 is involved in regulating programmed necroptosis across various organs, including the brain, kidney, liver, and heart in response to IRI-induced tissue damage and subsequent cell death [35, 53–56]. Inhibition of RIP1 has been demonstrated to reduce organ damage and prevent kidney failure in renal IRI models [54]. Yet, the use of a pan-caspase inhibitor did not effectively prevent IRI-mediated tissue damage [54]. Traditionally, apoptosis has been ascertained as the prevailing mechanism of cellular loss in numerous pathological conditions [62]. Nonetheless, endeavors to manipulate the apoptosis process through the use of a pan-caspase inhibitor have not been successful [63–65]. Various caspase inhibitors, serving as apoptosis blockers, have been investigated for the treatment of ocular diseases. However, apoptosis inhibitors cannot completely prevent the cell death process [63]. Furthermore, a lack of long-term protective effect of caspase inhibition was demonstrated in preclinical ocular models [64, 65].

RIP1 inhibitors have been raised as a promising therapeutic approach in protecting ocular degenerative diseases. In an experimental retinal detachment model, inhibiting RIP1 not only prevented photoreceptor cell death but also rescued the infiltration of CD11b-positive microglia and macrophages [66]. In addition, postmortem eyes from retinitis pigmentosa (RP) patients exhibited necrotic features together with higher levels of high mobility group-1 (HMGB1) protein in the vitreous of RP patients, reflecting necrotic cell death in RP [67]. To support this clinical observation, a study with an experimental RP model demonstrated that RIP1 activity is essential for the induction of cone necroptosis and microglia activation. [68]. RIP1-mediated retinal degeneration has also been reported in a mouse model of age-related macular degeneration (AMD) [69, 70]. In this AMD model, significant macrophage infiltration was observed, and RIP3-deficient mice exhibited reduced levels of proinflammatory mediators TNF and IL-6, as well as cell death [69]. In addition to RIP1 inhibitors, blocking TNF therapies have been shown to protect RGCs in ocular preclinical models. Previous studies demonstrated that anti-TNF treatments, employing etanercept or TNF-neutralizing antibodies, ameliorate ONC and IRI damage in the retina [45–48]. In addition, TNFR1 and TNFR2 knock-out mice exhibited significantly reduced neuronal loss following ONC [45, 71].

These IRI models in multiple organs share a common pathophysiology, which is tissue damage and excessive immune activation. Inflammation can be activated during ischemia and intensified upon reperfusion [72]. Macrophage infiltration into IRI lesions has been observed in multiple organs; this influx exacerbates the tissue damage via amplification of inflammatory cascades by promoting cytokine secretion and neutrophil recruitment, and by inducing cell death [73]. Notably, other groups have reported that a specific subset of microglia is regulated by RIP1 in the CNS [30, 31, 74]. This specific subpopulation of microglia called RIP1-regulated inflammatory microglia (RRIM) is distinguished by their high capability to produce proinflammatory cytokines and chemokines within the CNS [30, 31]. Levels of RRIM are upregulated in OPTN knock-out mice, and pharmacological inhibition of RIP1 effectively reduces the numbers of RRIM, underscoring that OPTN negatively regulates RIP1 [30, 31]. Together with RIP1, RIP3, and TNF serve as critical regulators of necroptosis, and neurodegenerative stress can elicit upregulation of both RIP3 and TNF [30, 75]. Especially, higher RIP3 signals are colocalized with high TNF levels in retinal degenerative disease models [75]. These findings are consistent with our data showing that human glaucomatous retinas exhibit higher levels of *RIP3* and *TNF*. Interestingly, OPTN-KO mice that show higher RIP1 kinase activity represent elevated RIP3 expression, proinflammatory cytokines, and axonal pathology phenotypes, suggesting that an abundance of RIP3 sensitizes to necroptosis [30].

Our research demonstrates the pivotal role of RIP1 in RGC function and survival which opens up the possibility for further investigations. This research holds significance since RIP1 inhibition may prevent tissue damage in other neurodegenerative disorders as well. Mitigating tissue damage by limiting cell death and restoring innate immune responses could inform future therapies and improve patient outcomes.

## Materials and methods

### Reagents and antibodies

Compounds BV6 (Genentech), Takinib (#6430, Tocris), Cycloheximide (#0970, Tocris), Emricasan (#S7775, SelleckChem), Doxorubicin (#2252, Tocris), Staurosporine (#1285, Tocris), GSK872 (#HY-101872, MedChemEspress), GNE684 (Genentech), and recombinant mouse TNF-alpha (#410-MT, R&D Systems) were used for the treatments of cellular assays. Immunoblot assays were performed with indicated primary antibodies: RIP1 (#53286, Cell Signaling Technology), Phospho-RIP1 (Ser166) (#31122, Cell Signaling Technology), RIP3 (#95702, Cell Signaling Technology), Phospho-RIP3 (Thr231/Ser232) (Genentech), MLKL (#37705, Cell Signaling Technology), Phospho-MLKL (Ser345) (#37333, Cell Signaling Technology), OPTN (#ab213556, Abcam or 100000, Cayman), actin (#ab6276, Abcam), and αtubulin (#ab7291, Abcam), BRN3α (#ab245230, Abcam), RBPMS (ABN1376, Millipore or #1832-RBPMS, PhosphoSolutions), SMI32 (#SMI-32P, BioLegend), IBA1 (#019-19741, WAKO), or NeuN (#ab104224, Abcam), Caspase-3 (#9962, Cell Signaling Technology), Cleaved caspase-3 (#9661L, Cell Signaling Technology), Neutrophil elastase (#M0752, DAKO) and CD-45 (#MCA345, Bio-Rad), Cleaved PARP-1 (#ab32064, Abcam).

### Human retina samples

San Diego Eye Bank provided the human retina tissue samples used in this study and confirmed that the donors provided their voluntary and informed consent for its use in research projects. All necessary permissions and authorizations have been obtained by the San Diego Eye Bank.

### Animal research

Animal experiments were approved by the Federal Food Safety and Veterinary Office of Switzerland. Experiments were conducted in strict adherence to the Swiss Animal Welfare Act (TSchG), Animal Protection Ordinance (TSchV), and Animal Experimentation Ordinance (TVV). *Ripk1*^*D138N*^ knock-in, *Ripk3*-KO, and *Mlkl*-KO C57BL/6 mice were generated at Genentech by CRISPR technology change references [76–79]. *Ripk1*^*D138N*^, *Ripk3*-KO, *Mlkl*-KO, and WT littermate mice were housed and maintained at Genentech in accordance with American Association of Laboratory Animal Care guidelines. All experimental animal studies with *Ripk1*^*D138N*^, *Ripk3*-KO, and *Mlkl*-KO mice were conducted under the approval of the Institutional Animal Care and Use Committees of Genentech Lab Animal Research. C57BL/6 mice at 8–12 weeks of age were ordered from Charles River or the Genentech Laboratory Animal Facility. Mice were acclimated to the new housing conditions for at least 7 days upon arrival. Animals were randomly assigned to each group. Samples were assessed in a blinded manner.

### Retinal ischemia–reperfusion injury model

Retinal ischemia–reperfusion injury (IRI) was induced as previously described [80]. Animals were anesthetized with a cocktail including 0.05 mg kg^−1^ fentanyl, 0.5 mg kg^−1^ medetomidine, and 5 mg kg^−1^ midazolam. One eye per animal was subjected to the IRI. The subjected eye was dilated by 5% tropicamide and anesthetized topically with 1% tetracaine. Increased intraocular pressure (IOP) was induced by placing a 0.9% NaCl reservoir 160 cm high connected to a 30-gauge needle. The velocity of 0.9% NaCl solution drops from tubing was approximately 22 drops per minute. The 30-gauge needle was placed carefully into the anterior chamber of the subjected eye without touching the lens. A pressure cuff added 30 mmHg of additional pressure on the 0.9% NaCl reservoir. Induced ischemia was monitored using an ophthalmoscope. After 45 min of IRI induction, mice were recovered from anesthesia by an antagonizing cocktail including 1.2 mg kg^−1^ naloxon, 2.5 mg kg^−1^ atipamezol, and 2.5 mg kg^−1^ flumazenil 2.5 mg kg^−1^. Eye ointment was applied after the operation. During the procedure, retinal ischemia was confirmed by observing whitening of the retina and reperfusion was reassured by observing the returning blood flow with an ophthalmoscope. The contralateral eye remained untreated. During the surgical procedure, the animals were kept on a heating pad to keep a stable body temperature. GNE684 was administered by oral gavage at 100 mg kg^−1^ twice per day during the induction of IRI.

### Optic nerve crush model

After intraperitoneal anesthesia, the optic nerve was accessed within the orbit via an incision in the tissue covering the superior border of the orbital bone. The superior orbital contents were dissected, and the rectus muscles were reflected laterally. To allow access to the optic nerve and surrounding dura mater sheath, the eye was rotated laterally by applying traction to the extraocular muscles. The optic nerve was exposed intraorbitally and crushed with self-closing forceps for 10 s at ~0.5–1 mm behind the optic disc. Eye ointment was applied after the operation.

### Adeno-associated virus 2 (AAV2) production

AAV2.7m8 capsid with CMV promoter was used to deliver payloads, including stuffer, OPTN-WT, and OPTN-E50K sequences. Briefly, the transfer plasmid containing the payloads with a proprietary Rep-cap plasmid and a helper plasmid encoding adenovirus genes (E4, E2A, and VA) responsible for AAV2 replication were co-transfected into HEK293T packaging cells. Following incubation, viral particles were collected from the cells, and concentrated using PEG precipitation. The viral particles underwent further purification and concentration using a gradient ultracentrifugation procedure for in vivo application. The AAV2 titer was determined using a qPCR-based method. Vector Builder was responsible for the production of AAV2.

### Adeno-associated virus 2 (AAV2) in vitro transduction

To test the transduction efficiency of the payload delivered by AAV2, different multiplicities of infection (MOI) conditions were tested in 661w cells. AAV2s carrying stuffer, OPTN-WT, and OPTN-E50K were transduced at 100 K MOI for further experiments. After 72 h of transduction, the level of transduction was confirmed by immunoblot assays.

### Intraocular adeno-associated virus 2 (AAV2) vector injection

Following an acclimatization period, the mice underwent anesthesia induction with 3% isoflurane in the induction box and maintained their anesthesia status through an inhalation mask. After a 15-min isoflurane induction, the eyes were flushed with sterile liquid, topically anesthetized, and lubricated. Pupil dilation was achieved using 1% tropicamide. Intravitreal injection (IVT) was then performed using a microinjection syringe pump and the Nanofil sub-microliter injection system (World Precision Instruments), equipped with a 34-gauge beveled needle, under a surgical operating microscope (Zeiss). A total of 1 × 10^13^ vector genomes (vg) were bilaterally injected into the vitreous of each mouse.

### Pattern electroretinogram (PERG)

Simultaneous PERG recording was completed with the CELERIS (Diagnosys LLC) system. Anesthetized mice were placed on a feedback-controlled heating pad to maintain body temperature at 37 °C. Eyes were dilated with 1% tropicamide (AKORN). Systane eye gel (Alcon) was added as needed to prevent corneal drying and possible eye irritation. The reference electrode was placed on the surface of the cornea of each eye. The pattern stimulator was placed on the contralateral eye for the simultaneous acquisition. The pattern remained at a contrast of 100% and a luminance of 50 cycles per degree per m^2^ and consisted of four cycles of black-gray elements, with a spatial frequency of 0.035 cycles per degree. Upon stimulation, the PERG signals were recorded by 200 consecutive sweep acquisitions. The low-frequency cut-off set was 0.125. Two consecutive recordings of 100 sweeps were averaged to achieve one readout. The first positive peak in the waveform was designated as P1 (P50), and the second negative peak as N2 (N95).

### Optomotor response (OMR)

Optomotor response was acquired by OptoDrum (STRIA-TECH) and analyzed with OptoDrum software (ver. 1.3.4). The mice were placed on a platform surrounded by four screens creating a box. The screens displayed a moving grid creating a virtual cylinder with varying frequencies at 99.72% of contrast. A camera at the top of the box tracked and monitored the head movement. The highest and lowest spatial frequency at which the mice could still track the moving grid were used to measure visual acuity. The right eye was represented by the grid rotated counterclockwise, while the left eye is represented by the grid rotated clockwise. Before beginning treatment, the baseline was examined, and follow-up measurements were carried out a day before the final experiment. A value of “−1” was interpreted as “efficient blind” status and plotted to the graph with value “0”.

### Optical coherence tomography (OCT)

To measure the average optical coherence tomography in vivo, PS-OCT, InVivoVue (ver. 2.4) Matlab image analysis Biopgene, Bioptigen AIM System (Leica) were performed for in vivo imaging. After intraperitoneal injection with an anesthesia cocktail including 0.05 mg kg^−1^ fentanyl, 0.5 mg kg^−1^ medetomidine, and 5 mg kg^−1^ midazolam for deep anesthesia, pupils were dilated with 5% tropicamide (AKORN). Images were obtained using the circular scanning mode with a diameter of 3.45 mm and centering on an optic disc (Heidelberg). The data were computed with Heidelberg software.

### Tissue processing

Enucleated eyes were drop fixed for overnight at 4 °C in 4% (v/v) paraformaldehyde (PFA) in PBS. Fixed eyes were dehydrated by using Tissue-Tek VIP 5 Jr (Sakura Finetek) and paraffin-embedded. Paraffin-embedded eyes were sectioned at 4 µm with Leica microtome (RM2255).

### Immunohistochemistry

Flat mounts of the retina were prepared for RGCs quantification. The eyes were harvested and incubated in cold 4% paraformaldehyde in PBS for 2 h at 4 °C. Then the retina was dissected and placed in blocking solution containing 5% BSA and 0.2% Triton X-100 and incubated for 1h at room temperature. The retina was incubated with BRN3α (Abcam), RBPMS (Millipore or PhosphoSolutions), SMI32 (BioLegend), IBA1 (WAKO), or NeuN (Abcam) primary antibodies diluted in blocking solution overnight at 4 °C. One day later, fluorophore-conjugated secondary antibodies were diluted in blocking solution and added to the flattened retinas for 2 h at room temperature. The retinas were washed three times with PBS each step and DAPI was added at the last washing step. The retinas were sealed with a coverslip and mounting medium (Sigma). Quantification of RGCs was conducted in retinal flat-mounts imaged by a ×20 objective lens. Sampling areas were 521 μm × 521 μm squares, located at the peripheral area 1 mm from the center of the retina. Six to eight images were taken in the peripheral area per retina and plotted. The quantification for SMI32-positive dead cells accounted only for the cell bodies smaller than 30 pixels and 25% brighter than the average intensity. The prepared tissue sections were digitally scanned using a VS120 Virtual Slide Microscope (Olympus) slide scanner, Zeiss LSM710, or Leica Thunder (Leica), and acquired images were analyzed with Zeiss Black (ver. 3.7) or Leica Application Suite X (ver. 3.7.6.25997). The number of BRN3α, RBPMS, IBA1, and SMI31 positive cells in flat-mounted retinas were analyzed by CellProfiler ver. 4.1.3 [81].

Cleaved caspase-3 immunohistochemistry was performed on paraffin-embedded sections of eye with the rabbit polyclonal anti-cleaved caspase-3 antibody (catalog no. 9661L, Cell Signaling Technology) at a concentration of 0.05 µg ml^−1^ on the Ventana Discovery XT platform (Roche Tissue Diagnostics). Antigen retrieval was performed with Ventana CC1 standard retrieval solution (Roche), and the Ventana Discovery Inhibitor was used as an enzyme block and bovine serum albumin was used as an immunoglobulin block. Immunolabeling was detected with the Rabbit OmniMap detection system and Discovery Purple was used as the chromogen. Slides were counterstained with hematoxylin. A tumor xenograft was used as a positive control and control tissue incubated with isotype-matched antibodies were used as a negative control.

Cleaved caspase-3 labeling was observed in the nucleus and/or cytoplasm of cells in the retina, particularly the inner nuclear layer, and uvea without defined morphologic features of apoptosis (e.g., pyknosis and karyorrhexis). Labeling was subjectively scored based on the extent of labeling in the uvea and retina according to the following criteria: (1) multifocal to segmental labeling, often in the iridociliary epithelium with limited labeling in the retina; (2) labeling of scattered cells throughout the inner nuclear layer of the retina or extensive labeling of the retina pigmented epithelium in addition to labeling in the iridociliary epithelium; (3) Similar to (2), but increased labeling in some components of the retina and/or uvea, particularly increased labeling of the iridociliary epithelium and/or choroid.

Neutrophil elastase (DAKO) and CD45 (Bio-Rad) immunohistochemistry were carried out on paraffin-embedded sections. The sections were deparaffinized by immersion in two changes of 100% xylene for 5 min each. Deparaffinized retina slides were then hydrated through sequential immersion in two changes of 100% ethanol for 3 min each, then in 95% ethanol for 1 min and 80% ethanol for 1 min. Post-dehydration, sections were rinsed thoroughly in distilled water. Antigen retrieval was performed by a pressure cooker for 30 min in a slide chamber containing Tris-EDTA buffer (pH 8.0). After completing the antigen retrieval step, sections were cooled down quickly with tap water and rinsed in PBS afterward. Slides were incubated with antibody-blocking buffer (5% BSA in PBS) for 1 h at room temperature, followed by primary antibody diluted in blocking buffer for overnight at 4 °C. Following primary antibody incubation, slides were rinsed with PBS twice and incubated with secondary antibodies diluted in blocking buffer for 2 h at room temperature.

### In situ hybridization

Processed tissue slides were baked for 1 h in a 55 °C oven and washed in xylene once before starting the procedure. The Ventana system was utilized to automate in situ hybridization. RNAscope 2.5 VS Probe- Hs-TNFA-C2 (#310429-C29, ACDBio), RNAscope 2.5 VS Probe-Hs-RIPK3 (#434668, ACDBio), and ACD RNA ISH Probe UBC (#312029, ACDBio) were used. Images were acquired with the slide scanner VS120 Virtual Slide Microscope (Olympus), and the signal was quantified by counting the number of signals per indicated area using HALO software (ver 3.2) or ImageJ.

### Gene editing

OPTN-KO-1 (sgRNA-1 and -2), OPTN-KO-2 (sgRNA-1 and -3), and OPTN-KO-3 (sgRNA-2 and -3) 661W cell lines were generated with two sgRNAs combination (Synthego) [sgRNA-1: AGAGAAAUCAGAAAAGCCAU, sgRNA-2: GAUUUGAGGAGCUGUCCGCC, and sgRNA-3: AGCUAUGAAAGGGCGAUUUG]. 661W cells were developed at and licensed by the University of Oklahoma, Baylor College of Medicine, and The University of Illinois at Chicago. All 661w lines were tested mycoplasma-free. The OPTN-KO-2 combination was used for the main experiment of the study. CRISPR/Cas9-mediated deletion of genes was performed by electroporation of Cas9 RNP in 661w. Briefly, 1 × 10^6^ cells were electroporated with 1 µg of recombinant Cas9 (ThermoFisher) complexed with gene-specific guide RNAs. Cells were suspended in 20 µL of SE Cell Line 16-well Nucleofector strips (Lonza) and electroporated using program CM-137 in 4D NucleofectorTM (Lonza). Following electroporation, cells were grown in T175 culture-treated plate (Corning) for an additional 5 days in complete media. Cells were collected from dishes and re-plated as required for in vitro assays in tissue culture-treated multi-well plates.

### OPTN-E50K cell line generation

All cell lines generated were only used for the experiment up to 15 passages after the nucleofections. To generate cell lines harboring *Optn* [NM_001356487] or *Optn* [148G>A (p.Glu50Lys)], PhiC31 integrase system was adapted (System Biosciences) following the manufacturer’s protocol. pFC-CAG-MCS-WPRE-pA-SV40-Neo PhiC31 Donor Vector (FC600A-1) carrying OPTN and OPTN-E50K coding sequences were produced by GeneScript. 1:3 molar ratio of PhiC31 integrase expression vector (FC200A): donor vector was electroporated into CRISPR-engineered OPTN-deficient cells with SE Cell Line 16-well Nucleofector strips (Lonza) using 4D NucleofectorTM (Lonza). After 10 days of neomycin antibiotic selection, cells were ready to use.

### Cell death assay

The culture medium from 96-well plates was analyzed for LDH release with the Cytotoxicity Detection Kit (LDH) (Roche). The standard protocol assays were performed according to the manufacturer’s instructions.

### Immunoblotting

Cellular lysates were collected in RIPA lysis buffer (ThermoFisher) supplemented with 1 mM PMSF and protease inhibitor cocktail (Cell Signaling Technology). Collected cell lysates were further centrifuged at 14,000 rpm for 20 min at 4 °C. The supernatants were recovered and protein concentrations were measured by the BCA Protein Assay Kit (ThermoFisher) or Direct Detect Spectrometer (Millipore) following the manufacturer’s protocol. Capillary-based immunoblotting was performed with Peggy Sue or WES following the manufacturer’s instruction (Protein Simple). Immunoblot assays were performed with indicated primary antibodies: RIP1 (Cell Signaling Technology), Phospho-RIP1 (Ser166) (Cell Signaling Technology), RIP3 (Cell Signaling Technology), MLKL (Cell Signaling Technology), Phospho-MLKL (Ser345) (Cell Signaling Technology), OPTN (Cell Signaling Technology, Abcam, and Cayman), actin (Abcam), and tubulin (Abcam). All the original blots are represented in a Supplementary File.

### Quantitative RT-PCR

Total RNA was extracted using an RNeasy Mini Kit (QIAGEN) following the manufacturer’s guide and quantified in a NanoDrop 8000 (ThermoFisher). Extracted RNA was used for synthesizing cDNA using SuperScript VILO MasterMix (Invitrogen). Quantitative RT-PCR was carried out using TaqMan Fast Advanced MasterMix (ThermoFisher) and genes were amplified using TaqMan Assays (ThermoFisher): Optn (Mm01333245_m1), Rplp0 (Mm00725448_ss1), Tnf (Mm00443258_m1), Il1α (Mm00439620_m1). For the tissue analysis, the investigator was blinded to group allocation during the experiment.

### Statistical analysis

All statistical analyses were performed in GraphPad Prism ver. 9.1.0 software. For group comparisons, two-way ANOVA was used. Data from two groups were analyzed using two-tailed Student’s *t* test. Data with three or more groups were analyzed with a one-way ANOVA test that was performed for multiple comparisons. Differences in means across multiple groups with multiple measurements over time were analyzed using two-way ANOVA. In all analyses, a minimum *P* < 0.05 was considered statistically significant. All experiments were repeated multiple times, and at least twice.

## Supplementary information

Supplemental Material

## Data Availability

All data reported in this study will be shared by the lead contact upon request.

## Materials availability

All materials reported in this study will be shared by the lead contact upon request.
