# Supplementary material for: RIP1 inhibition protects retinal ganglion cells in glaucoma models of ocular injury
Source: Cell Death Differ. 2024 Oct 24;32(2):353–68. doi: 10.1038/s41418-024-01390-7 (PMC11802773; doi:10.1038/s41418-024-01390-7)

RIP1 inhibition protects retinal ganglion cells in glaucoma models of ocular injury

Supplementary Information

Suppl Figure 1. RIP1 inactivation or RIP3 or MLKL ablation protects the RGC in an ONC glaucoma preclinical model.

(**A**) Schematic representation of the experimental setup for animal models. (**B**) Optic nerve crush (ONC) was induced in wild-type and RIP1-KD mice, and retinas were subjected to further evaluation at 7 days post-ONC. (**C**) Schematic representation of sampling method for imaging retinal whole-mounts. Square box is a representation of imaging area. (**D**) Representative images of the RGC layer (GCL) from ONC glaucoma model at 7 days post-ONC and (**E**) quantification of IBA1 and (**F**) NeuN-positive cells in WT (n=7), RIP3-KO (n=6), and MLKL-KO (n=10) mice. (**G**) Representative graph of pattern electroretinogram (PERG) recording from each condition, including intact retina, contralateral, and 7 days post-ONC. (**H**) Quantification of PERG recording following ONC in WT (n=7), RIP3-KO (n=6), and MLKL-KO (n=10). (**I**) Visual acuity test with optomotor response (OMR) evaluation at 6 days post-ONC in WT (n=6), RIP3-KO (n=6), and MLKL-KO (n=8). (**J**) Quantification of the thickness of the retina measured by optical coherence tomography (OCT) at 7 days post-ONC in WT (n=5), RIP3-KO (n=3), and MLKL-KO (n=6). (**K**) A summary of statistical evaluation of all functional and structural readouts of ONC models. Arrow point up indicates increase and arrow pointing down indicates decrease; n/s non-significant. GCL: ganglion cell layer; INL: inner nuclear layer; ONL: outer nuclear layer. Scale bar = 50 µm. Two-way ANOVA. *$P < 0.05$, **$P < 0.01$, ****$P < 0.0001$.

Suppl Figure 2. RIP1 inhibition mitigates IRI-induced retinal damage in the preclinical glaucoma model.

(**A**) Timeline schematic illustration of the ischemia-reperfusion injury (IRI) model for 2 and 7 days post-injury conditions. GNE684 administration time points for the 2D post-IRI condition are represented by blue triangle marks. (**B**) Representative images from the retinal flat mount of each condition, including untreated

(intact retina), sham, and at 2 days post-IRI. Scale bar = 100 μm. (**C**) Representative images from the retinal flat-mount of each condition, including sham and sham with GNE684 100 mg kg^{-1} PO twice per day. (**D-E**) Quantification of the number of RGCs. (**D**) BRN3α and (**E**) RBPMS-positive cells were counted as RGCs. n=5 mice per group. ****P < 0.0001.

Suppl Figure 3. Genetic ablation of RIP3 or MLKL ameliorates IRI-induced retinal damage.

(**A**) Representative images of the RGC layer (GCL) from IRI glaucoma model at 7 days post-IRI and (**B**) quantification of IBA1 and (**C**) NeuN-positive cells in WT (n=9), RIP3-KO (n=7), and MLKL-KO (n=8) mice. (**D**) Representative graph of pattern electroretinogram (PERG) recording from each condition, including intact retina, contralateral, and 7 days post-IRI. (**E**) Quantification of PERG recording following IRI in WT (n=13), RIP3-KO (n=7), and MLKL-KO (n=19). (**F**) Visual acuity test with optomotor response (OMR) evaluation at 6 days post-IRI in WT (n=9), RIP3-KO (n=7), and MLKL-KO (n=18). (**G**) Quantification of the thickness of the retina measured by optical coherence tomography (OCT) at 7 days post-IRI in WT (n=5), RIP3-KO (n=4), and MLKL-KO (n=8). (**H**) A summary of statistical evaluation of all functional and structural readouts of IRI models. Arrow point up indicates increase and arrow pointing down indicates decrease; n/s non-significant. GCL: ganglion cell layer; INL: inner nuclear layer; ONL: outer nuclear layer. Scale bar = 50 μm. Two-way ANOVA. *P < 0.05, **P < 0.01, ***P < 0.001, ****P < 0.0001.

Suppl Figure 4. OPTN deletion and OPTN-E50K mutation do not affect RIP1-independent apoptosis.

(**A**) Illustrative representation of four different sgRNAs targeting OPTN. (**B**) Immunoblots confirming deletion of OPTN in 661w cells with indicated sgRNA combinations. (**C**) OPTN-KO lines generated by different combinations of sgRNA were stimulated with the necroptosis inducer TBE (100 ng mL^{-1} TNF, the indicated doses of BV6 and 10 μM Emricasan) for 24 h. Relative cell death was measured by LDH release. (**D-E**) OPTN-KO cell line was reconstituted with PhiC-OPTN-WT or PhiC-OPTN-E50K. Generated cell lines were analyzed by qPCR (**D**) mRNA level of *Optn* and (**E**) OPTN immunoblot assay.

(**F**) OPTN-KO cell line was reconstituted with PiggyBac (PB)-OPTN-WT and PB-OPTN-E50K. Cells were treated with necroptosis inducer TBE (20 ng mL^{-1} TNF, 2.5 μM BV6 and 10 μM Emricasan) alone or with 20 μM GNE684 for the indicated time. Immunoblot assay was perform for indicated proteins. Immunoblot assay of parental cells treated with (**G**) 2 μM Staurosporine, (**H**) 10 μM Doxorubicin or (**I**) TCHX (100 ng mL^{-1} TNF and 100 μg mL^{-1} Cycloheximide) alone or with 20 μM GNE684 for the indicated time. (**J-L**) Parental and OPTN-KO or (**M-O**) OPTN-WT and OPTN-E50K cells were treated with staurosporine, doxorubicin, or TCHX. The cell death ratio was evaluated by the LDH assay. Data represented as Mean ± SD. n/s non-significant.

Suppl Figure 5. AAV2 transduces retinal ganglion cell layers.

In vivo evaluation of AAV2-Stuffer, AAV2-OPTN-WT, and AAV2-OPTN-E50K transduced eyes with optical coherence tomography (OCT) and fluorescent angiography. 4 weeks of AAV2 transduction. (**A**) Representative fluorescent angiography images from AAV2-Stuffer, AAV2-GFP transduced retinas. Stuffer- and GFP-transduced retinas were prepared in flat-mount on the slide. Representative images were visualized with confocal microscopy. (**B**) Representative images from stuffer- and GFP-transduced retina cross-sections. Each cross-section was visualized with confocal microscopy. GCL: retinal ganglion cell layer; INL: inner nuclear layer; ONL: outer nuclear layer. (**C**) Representative images of GFP- and Stuffer-transduced retina. Immunostaining for indicated proteins. Visualized with confocal microscopy. Scale bar = 100 μm. (**D**) The level of OPTN and actin were measured in stuffer, OPTN-WT, and OPTN-E50K transduced retinas. (**E**) Immunoblot data of retinal cells transduced with AAV2 expressing OPTN-WT and OPTN-E50K. AAV2s carrying stuffer, OPTN-WT, and OPTN-E50K were transduced into 661w parental cell line for 72 h. After transduction, cells were treated with TBE (100 ng mL^{-1} TNF, 2.5 μM BV6 and 10 μM Emricasan) alone or with 20 μM GNE684 for 2 h. Immunoblot assay was performed for indicated proteins.

Suppl Figure 6. Control staining for human retinas.

In situ hybridization images of human non-glaucomatous (n = 4) and glaucomatous (n = 8 eyes) human retinas for *ubiquitin C* (UBC) (purple) and Hoechst nucleus counterstaining (blue). 40x Olympus scanner microscope. Scale bar = 50 μm.

Suppl Figure 7. Immunohistochemistry of TNF, IBA1, and RIP3 in human glaucomatous and non-glaucomatous retinas.

Representative images of human non-glaucomatous (n = 3 eyes) and glaucomatous (n = 6 eyes) retinas were included in the study (**A**) Post-mortem glaucomatous and non-glaucomatous human retinas were co-labeled with IBA1 (green) immunolabeling and in situ hybridization of *TNF* (pink). Sample ID 140001, 140016, 140099 and 150001 were used as representative images on Figure 7A. Scale bar = 50 μm. (**B**) *In situ* hybridization images for *TNF* (pink) and *RIP3* (green) indicated with arrows from post-mortem human glaucomatous patients and non-glaucomatous retinas. 40x Olympus scanner microscope. Sample ID 140001 and 140149 were used as representative images on Figure 7C. Scale bar = 50 μm and 20 μm. (**C**) Representative images of post-mortem glaucomatous and non-glaucomatous human retinas were labelled with Neutrophil elastase (green) indicated by white arrow heads and DAPI (blue). Sample ID AE02120 and 150001 were used as representative images on Figure 7G. Scale bar = 100 μm. (**D**) Representative images of post-mortem glaucomatous and non-glaucomatous human retinas were labelled with CD45 (red) and DAPI (blue). Sample ID 140015 and 150001 were used as representative images on Figure 7I. Scale bar = 100 μm.

Suppl Table 1. Information of human retina tissue samples.

Clinical information includes ocular history for each donor, age, a summary of medical history, sex, and ocular history records before death.

Suppl. Figure 1

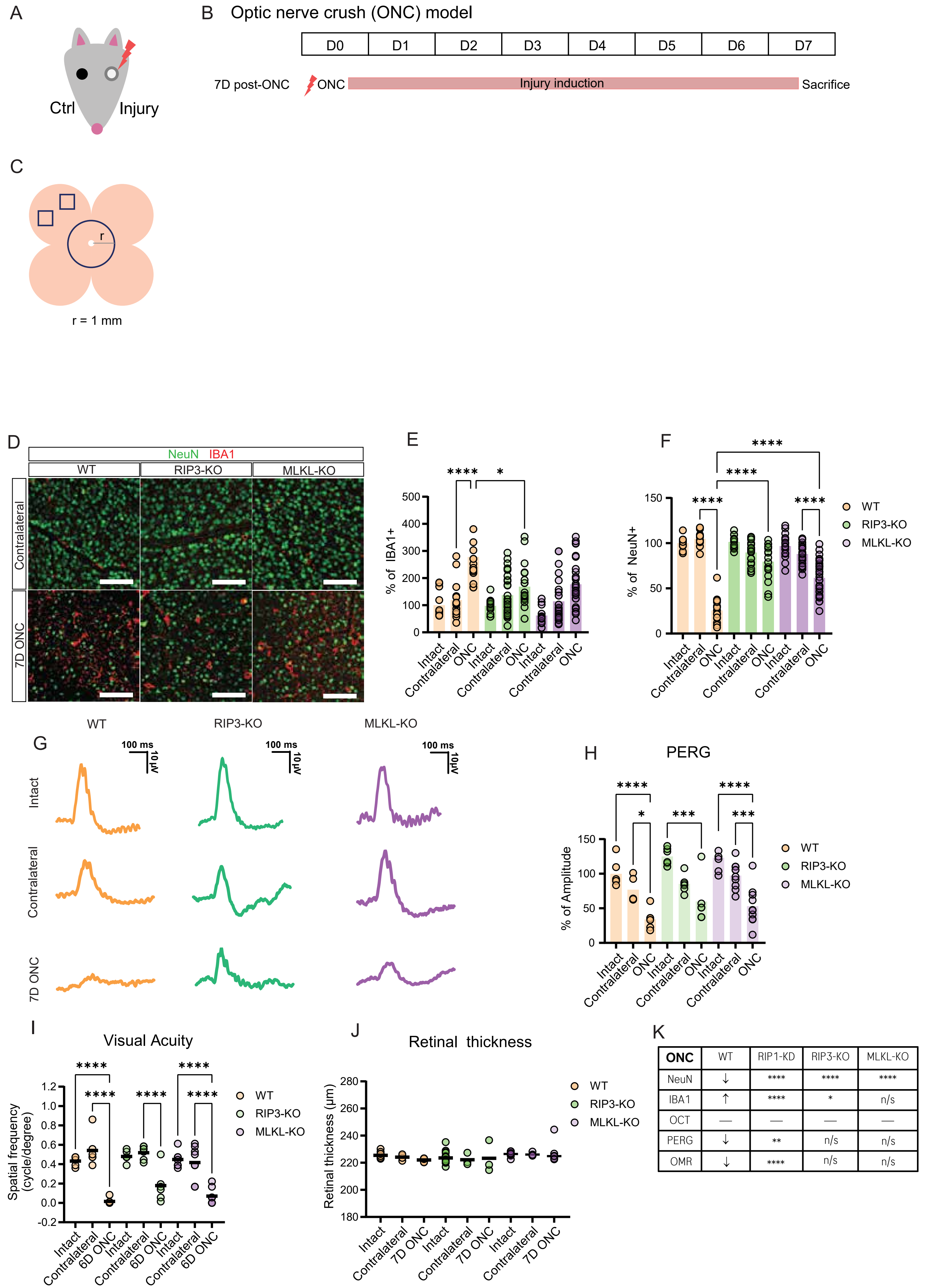

ONC	WT	RIP1-KD	RIP3-KO	MLKL-KO
NeuN	↓	****	****	****
IBA1	↑	****	*	n/s
OCT	—	—	—	—
PERG	↓	**	n/s	n/s
OMR	↓	****	n/s	n/s

Suppl. Figure 2

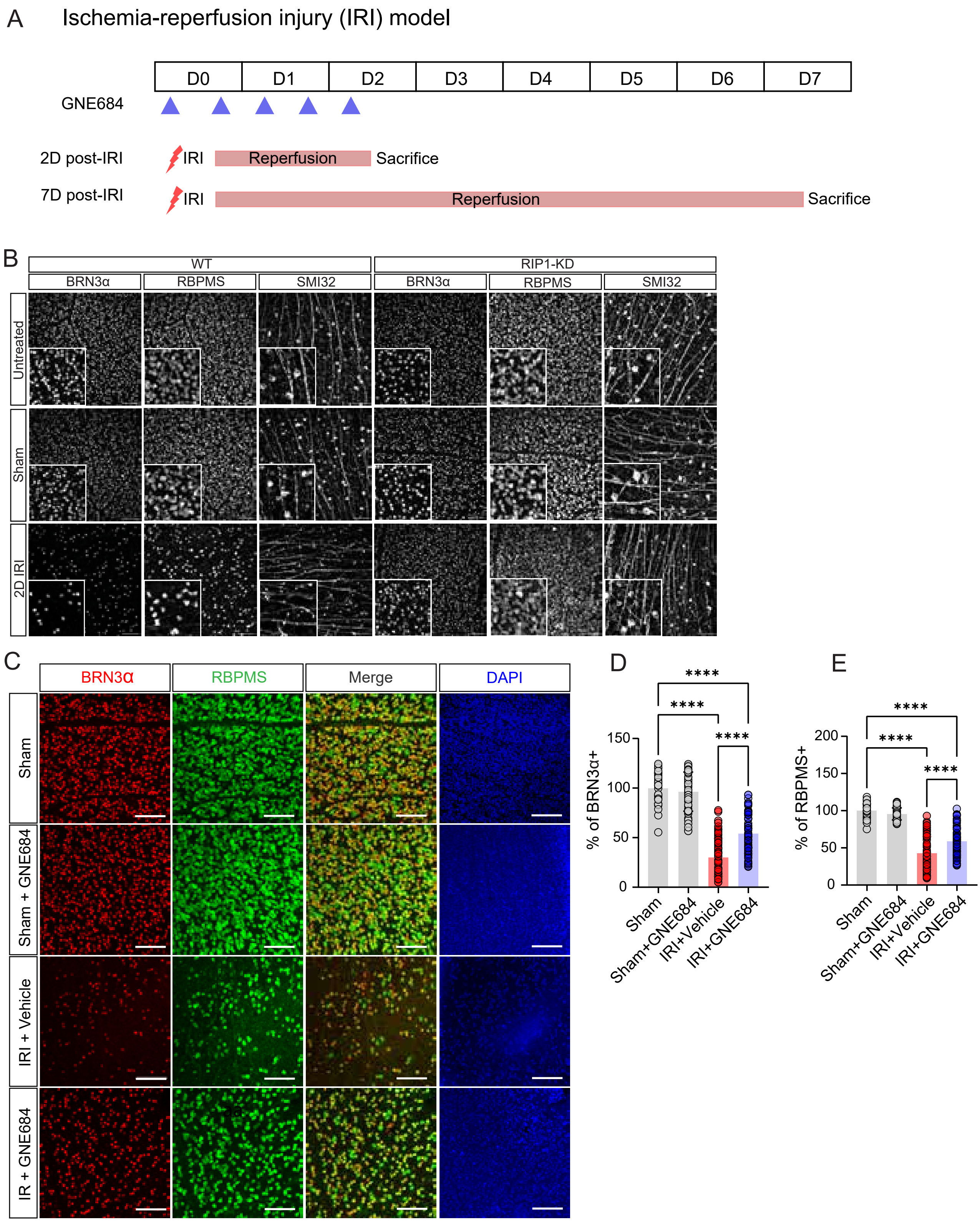

Suppl. Figure 3

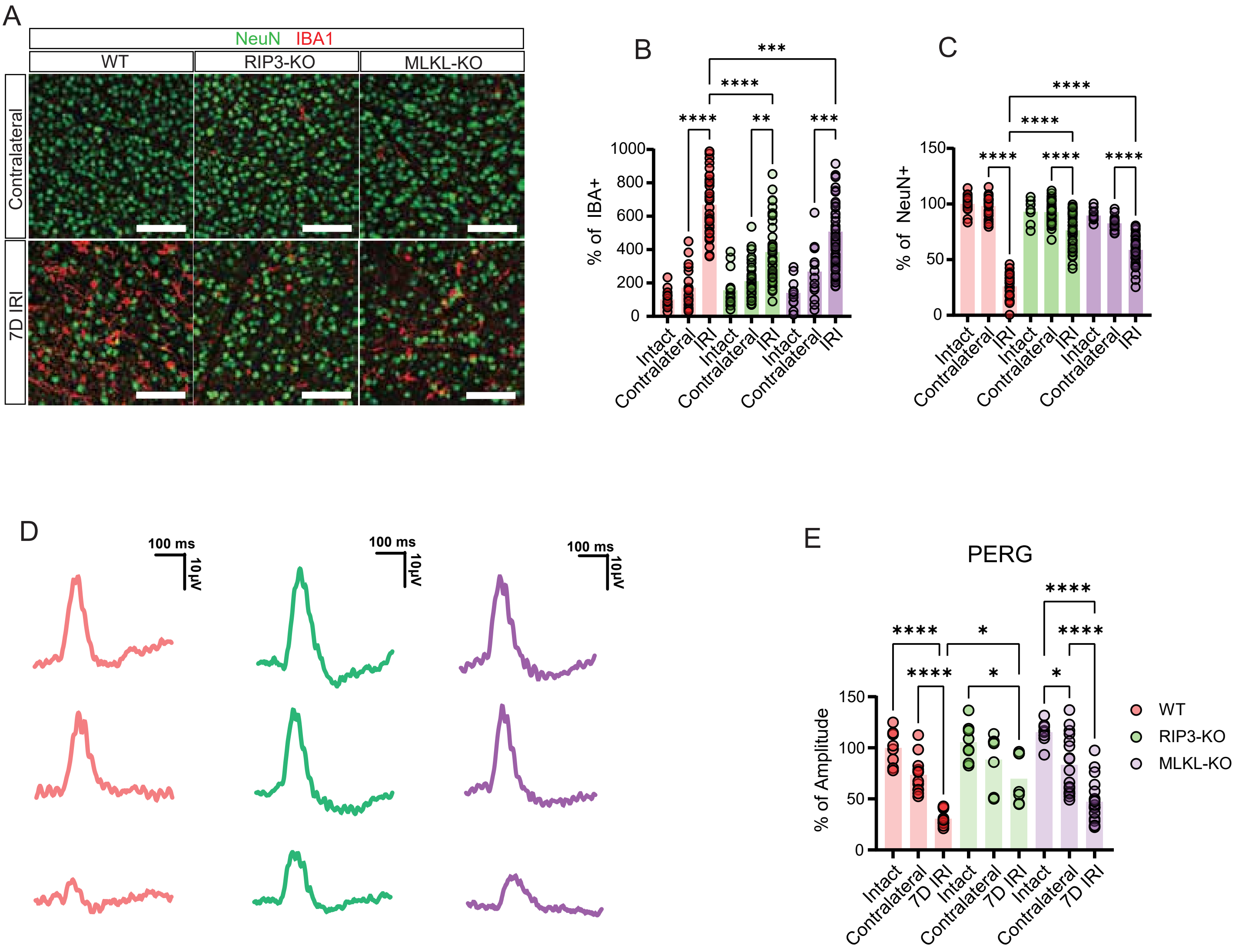

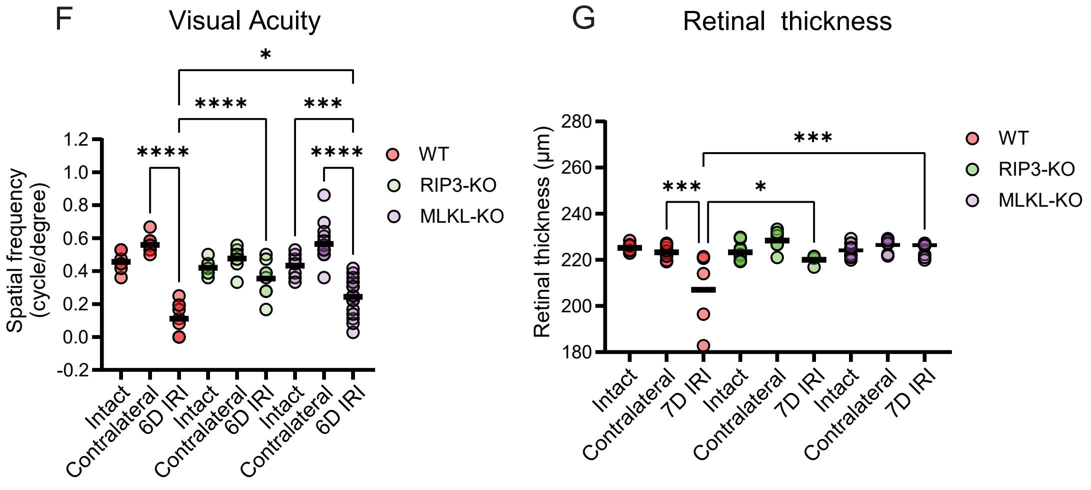

H

IRI	WT	RIP1-KD	RIP3-KO	MLKL-KO
NeuN	↓	****	****	****
IBA1	↑	****	****	***
OCT	↓	*	*	***
PERG	↓	****	*	n/s
OMR	↓	****	****	*

Suppl. Figure 4

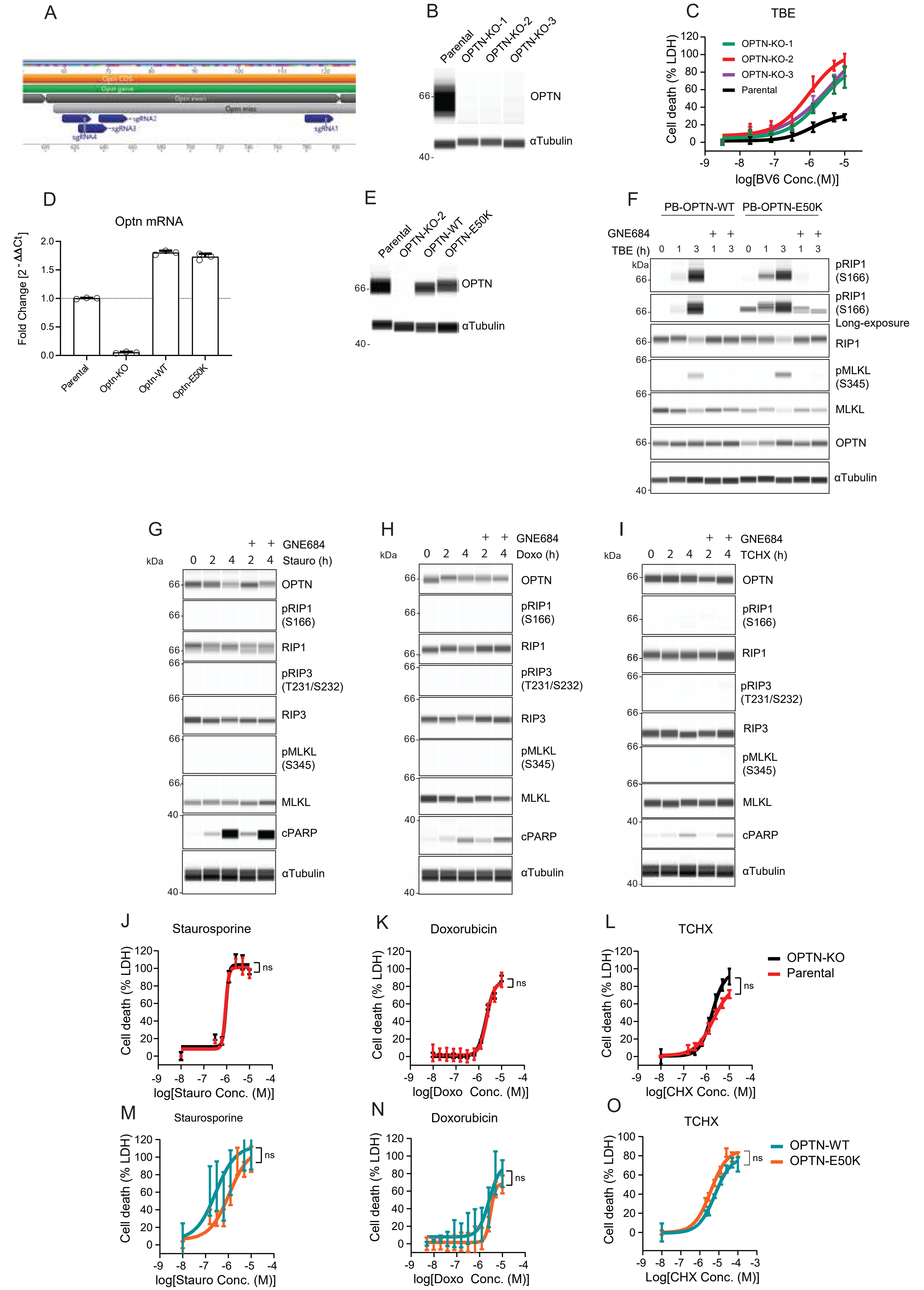

Suppl. Figure 5

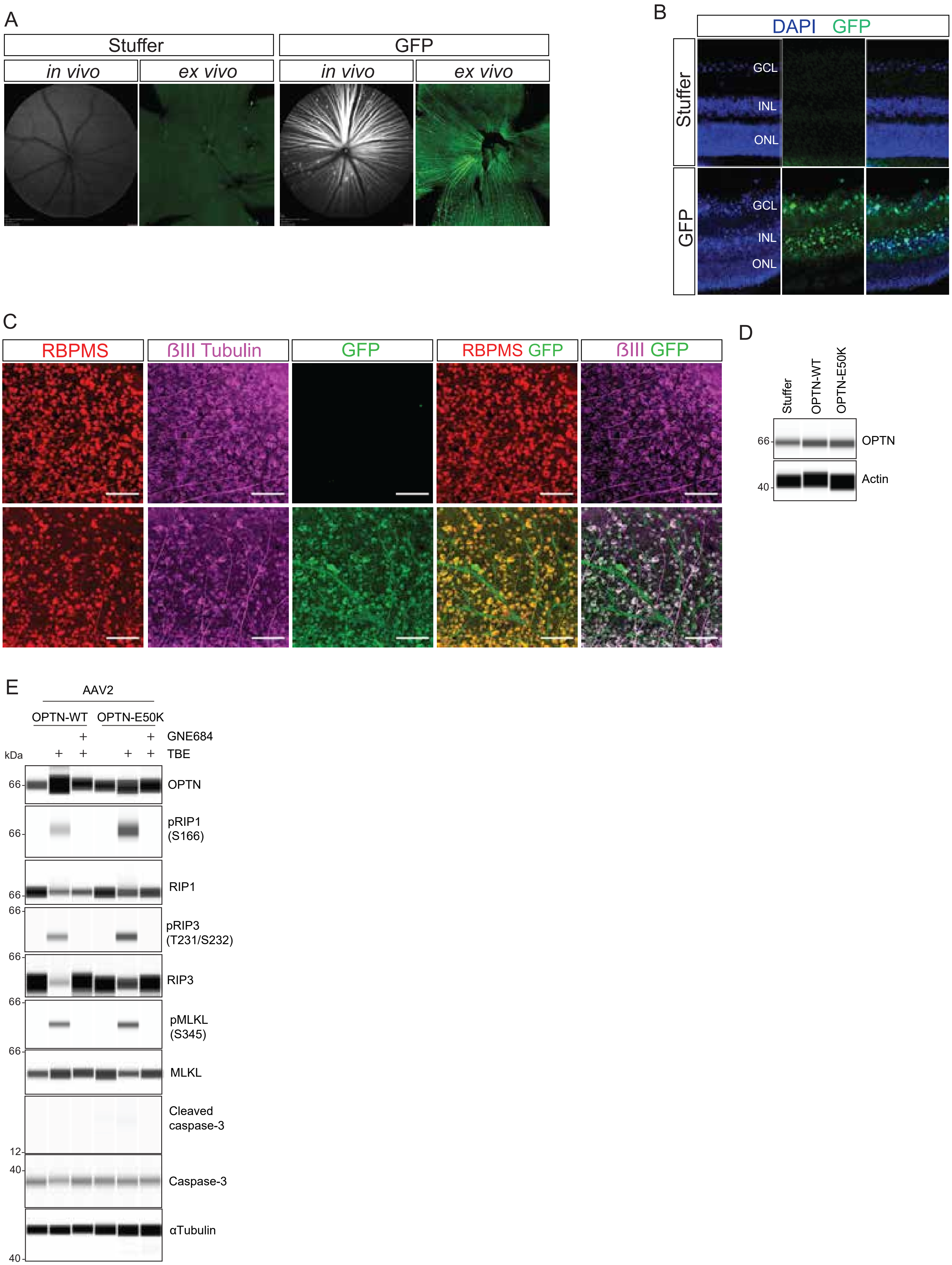

Suppl. Figure 6

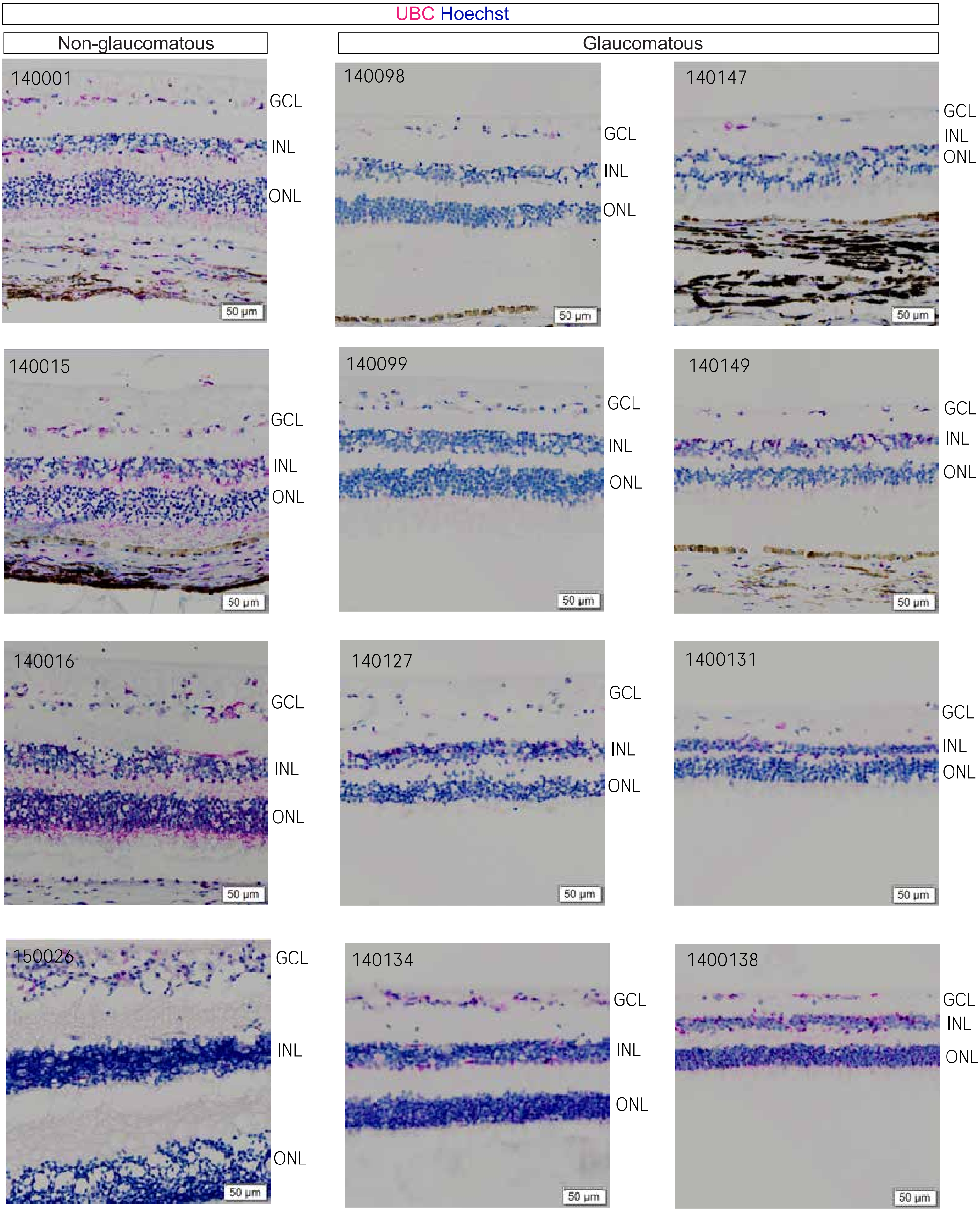

Suppl. Figure 7A-B

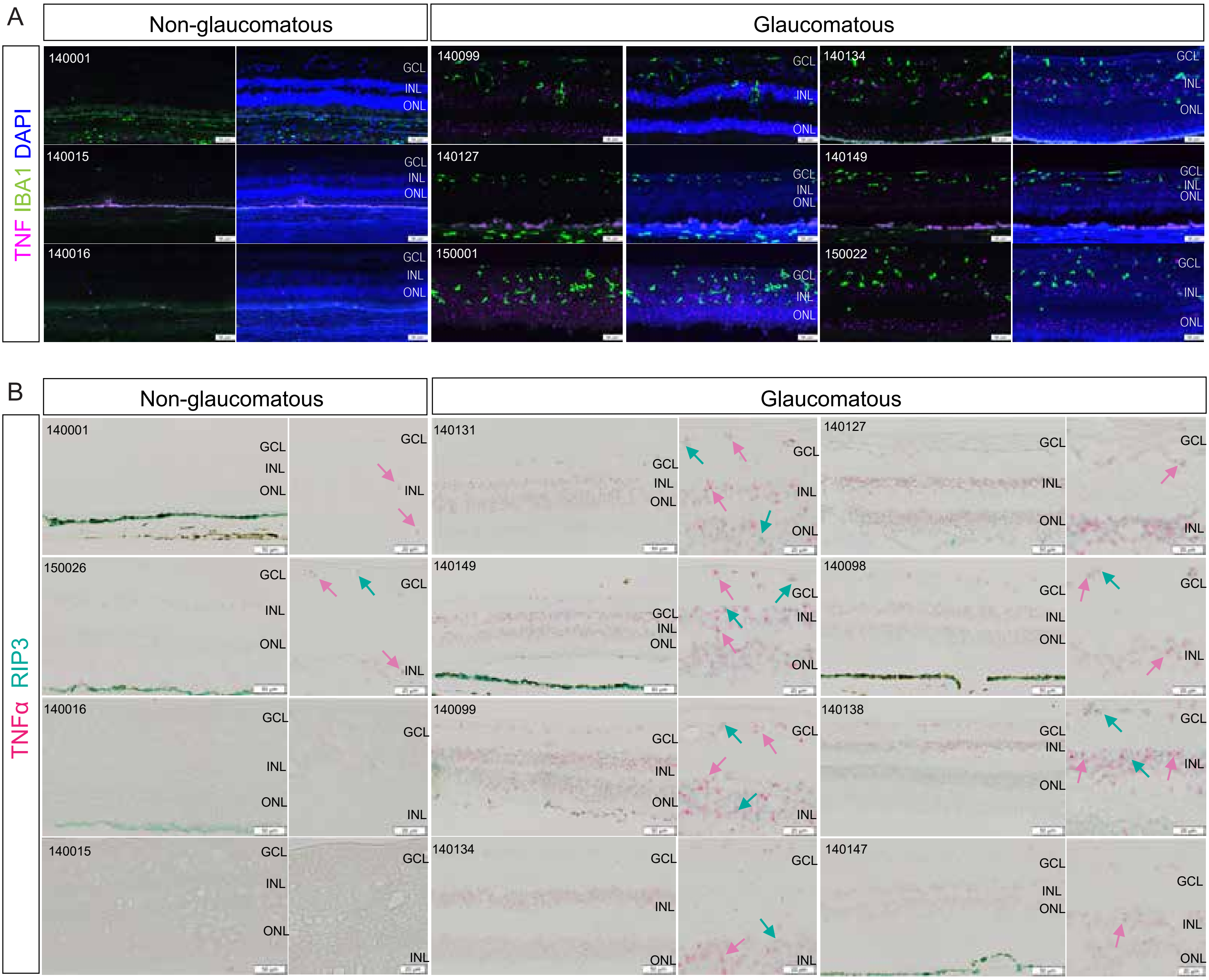

Suppl. Figure 7C

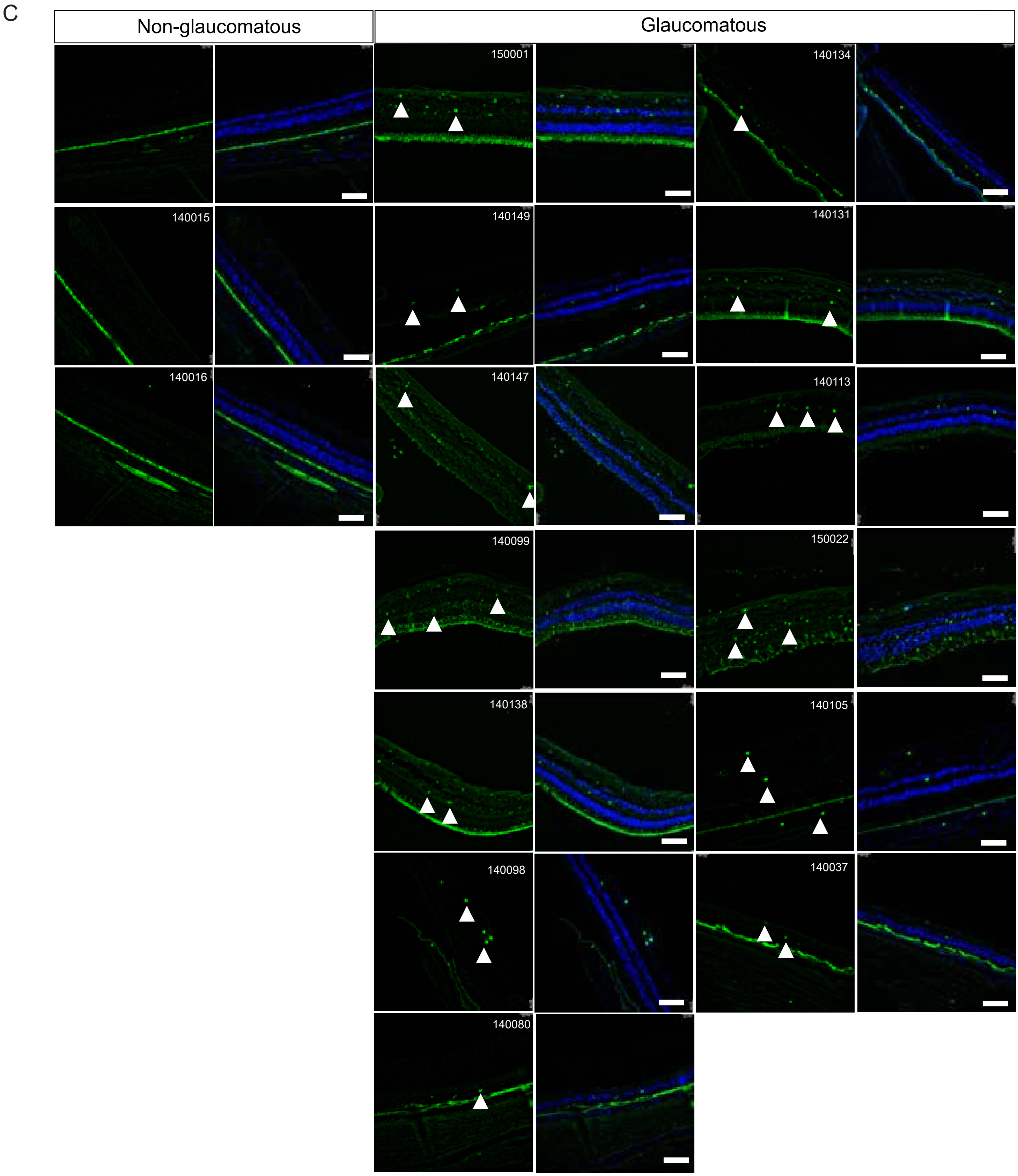

Suppl. Figure 7D

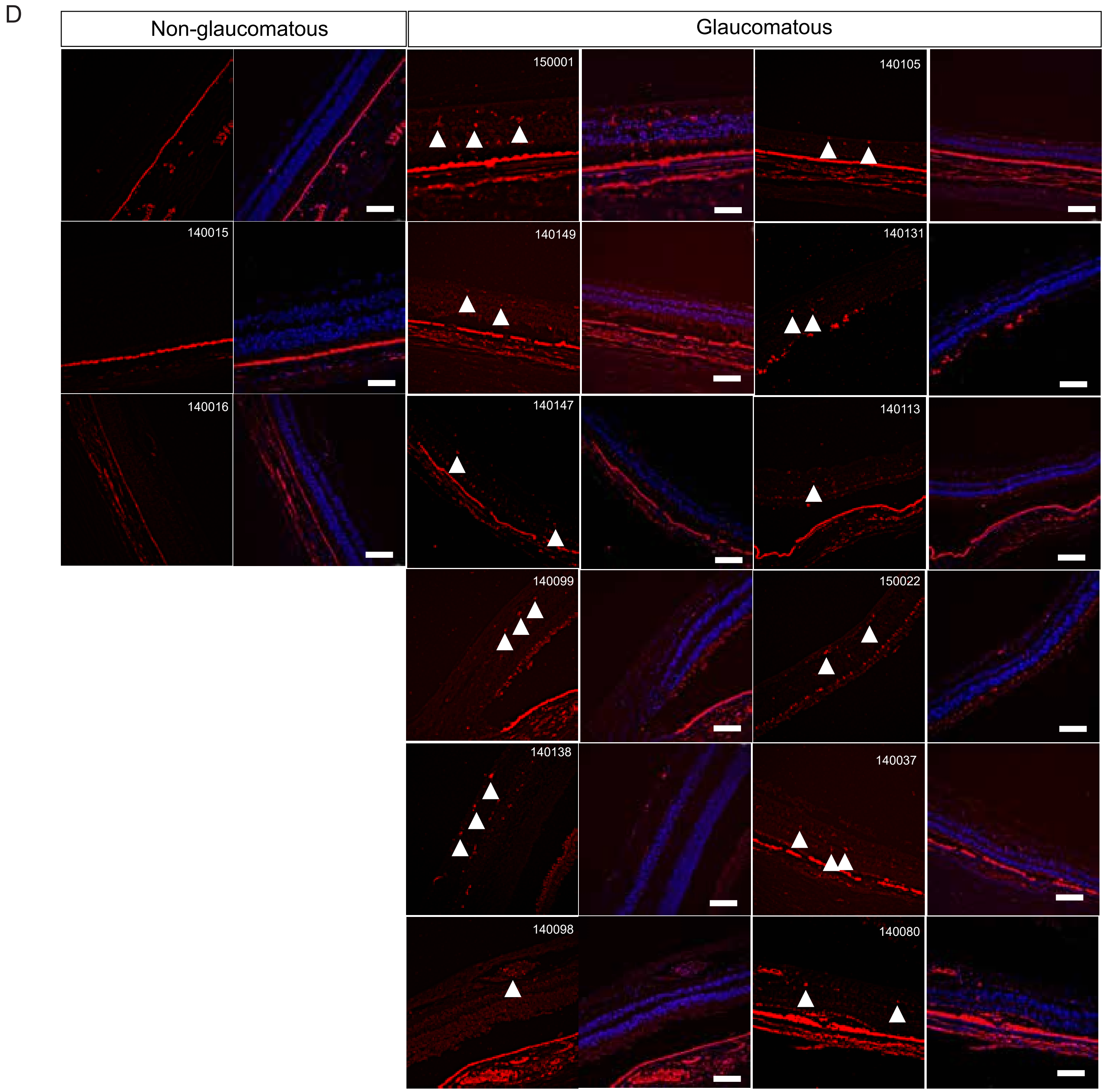

Suppl. Table 1

Donor	Condition	ID	Ocular history	Age	Summary of medical history	Sex
1	Non-glaucomatous	140001	Cataract surgery	83	N/A	M
2	Non-glaucomatous	140015	Posterior capsule,<br>Intraocular lens,<br>Oculus uterque	82	N/A	F
3	Non-glaucomatous	140016	Cataract surgery	83	N/A	M
4	Non-glaucomatous	150026	Posterior capsule,<br>Intraocular lens,<br>Oculus uterque	82	Metastatic colon cancer	F
5	Non-glaucomatous	AE02120	Normal	59	COPD<br>Type II diabetes<br>GERD<br>CAD<br>NSCLC<br>Screened negative for Covid-19<br>Previous smoker: 80 pack zears<br>Past alcohol abuse<br>Tympanic membrane repair<br>Bilateral carpal tunnel releases<br>CABG	M
6	Glaucomatous	140099	POAG glaucoma,<br>Posterior capsule,<br>Intraocular lens,<br>Oculus Uterque	88	Pneumonia<br>Dementia<br>Leukemia	M
7	Glaucomatous	140098	Glaucoma	82	Sepsis	F
8	Glaucomatous	140127	AMD,<br>Cataract,<br>Glaucoma	92	Sepsis	F
9	Glaucomatous	140131	Cataract surgery,<br>Glaucoma,<br>AMD	91	Lung cancer	M
10	Glaucomatous	140134	Glaucoma,<br>Posterior capsule,<br>Intraocular lens,<br>Oculus uterque	89	Gastrointestinal bleed	M
11	Glaucomatous	140138	Glaucoma	86	Gastrointestinal bleed<br>Dementia	M
12	Glaucomatous	140147	Glaucoma	91	Myocardial infarction	F
13	Glaucomatous	140149	Glaucoma,<br>Cataract surgery	87	Congestive heart failure	F

14	Glaucomatous	150001	Posterior capsule,<br>Intraocular lens,<br>Oculus uterque,<br>AMD	97	Chronic obstructive pulmonary disease	F
15	Glaucomatous	150022	Glaucoma,<br>Diabetic retinopathy	64	Leukemia<br>Myelodysplastic syndrome<br>Coronary artery disease<br>Hypertension.	F
16	Glaucomatous	140113	Glaucoma	82	Pneumonia<br>A history of lung adenocarcinoma with metastasis to brain and myelogenous leukemia<br>A right cerebellar infarct and stable edema of the right occipital lobe	F
17	Glaucomatous	140138	Glaucoma	86	Smoked from 18 to 46 and was smoke-free for 40 years<br>Dementia<br>Prerenal azotemia<br>Bypass surgery on left lower extremity due to vascular insufficiency<br>Prostate hypertrophy	M
18	Glaucomatous	140105	Glaucoma	72	Hypertension<br>Anemia and leukemia<br>Lung cancer<br>Pneumonia<br>Chronic heart failure<br>Chronic GI bleed<br>Rheumatoid arthritis	F
19	Glaucomatous	140037H	Glaucoma<br>AMD	89	Orthostatic hypotension<br>Smoked - pack a day for 30yrs.<br>Idiopathic parkinson's disease<br>Myocardial infarction<br>Mesenteric ischemia<br>Inflammation of the gall bladder<br>Atrial fibrillation<br>Pleural effusions<br>Anxiety<br>Anemia<br>Leukocytosis<br>Hyponatremia.	F
20	Glaucomatous	140080A	Glaucoma	90	Hypothyroidism<br>Osteoporosis<br>Coronary artery disease<br>Renal failure	F

Figure 5B

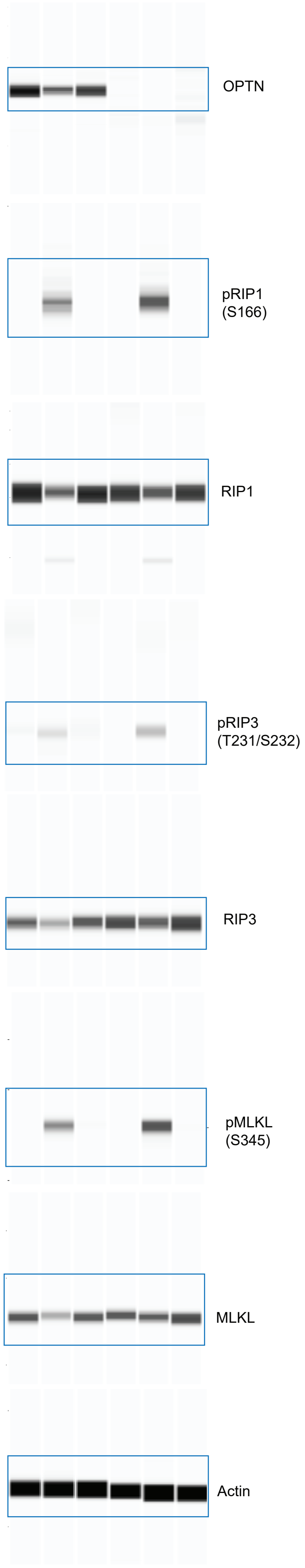

Figure 5D

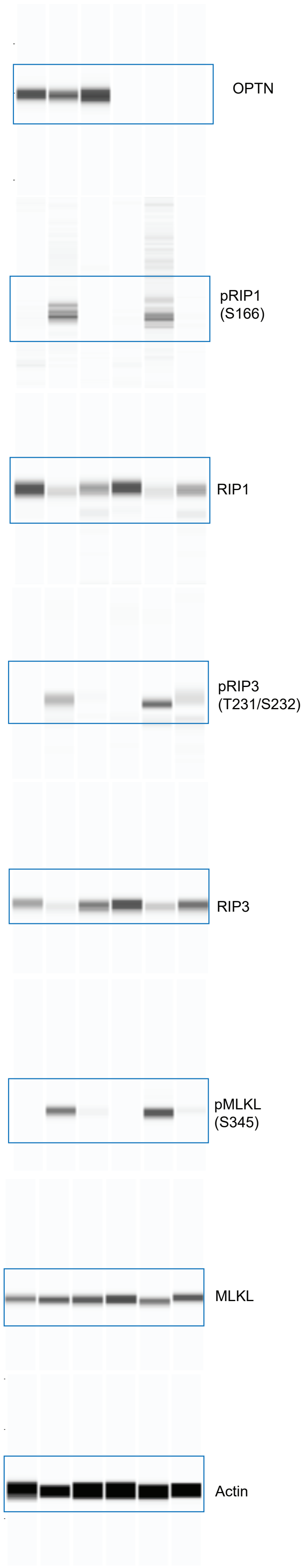

Figure 5F

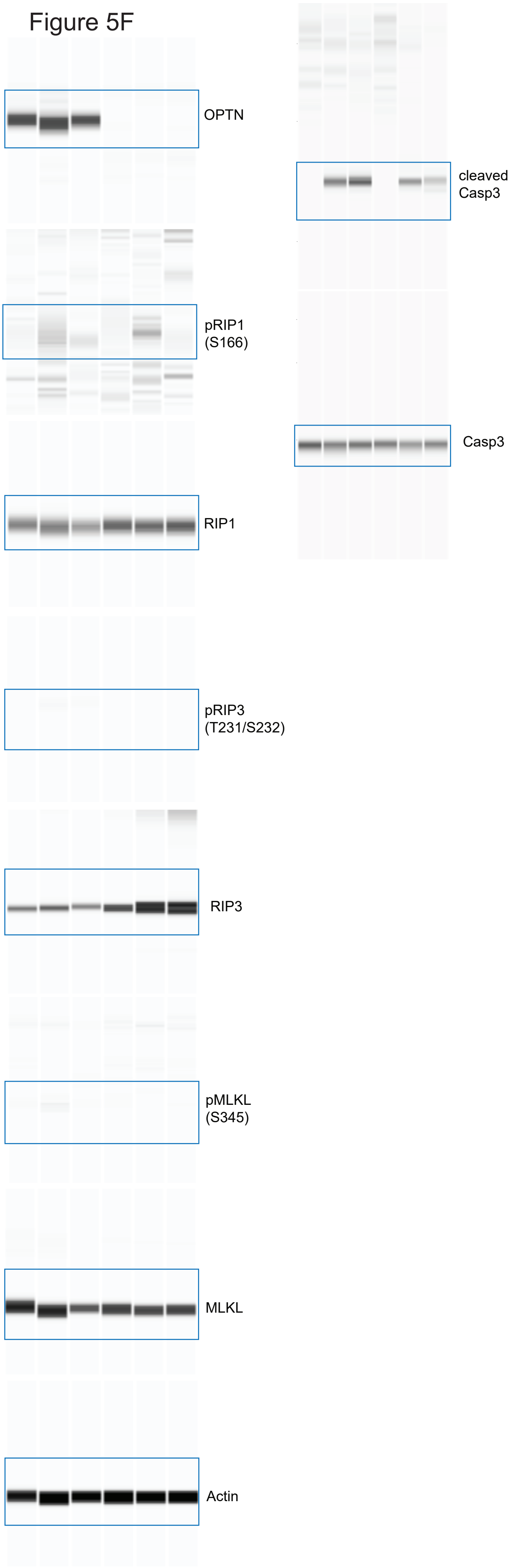

Figure 5H

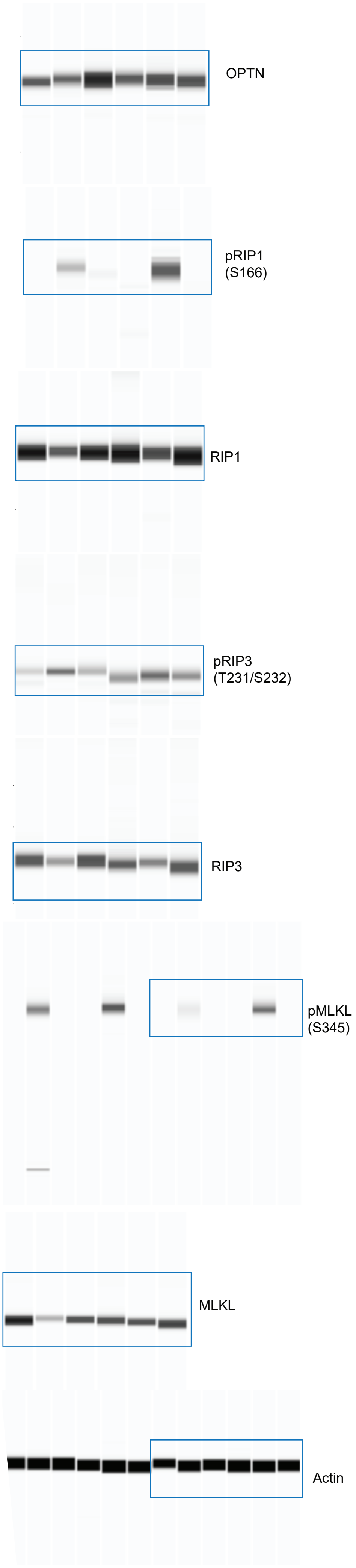

Figure 5J

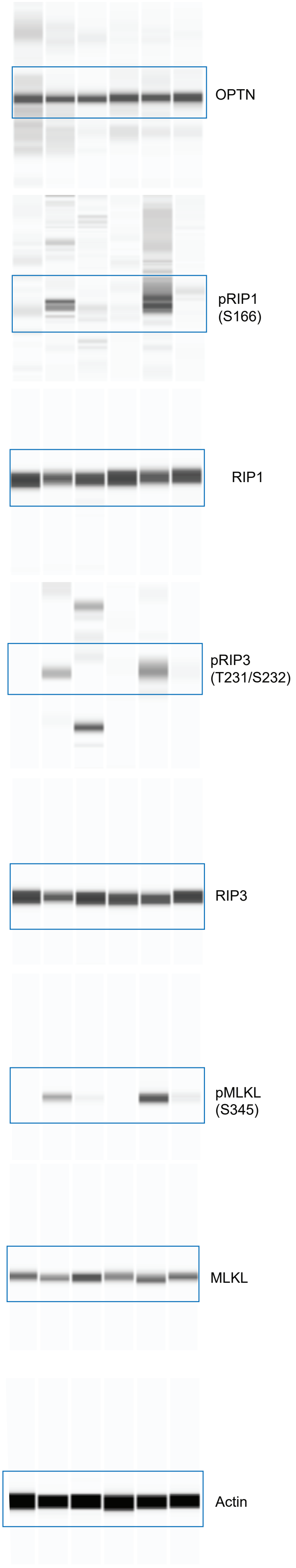

Figure 5L

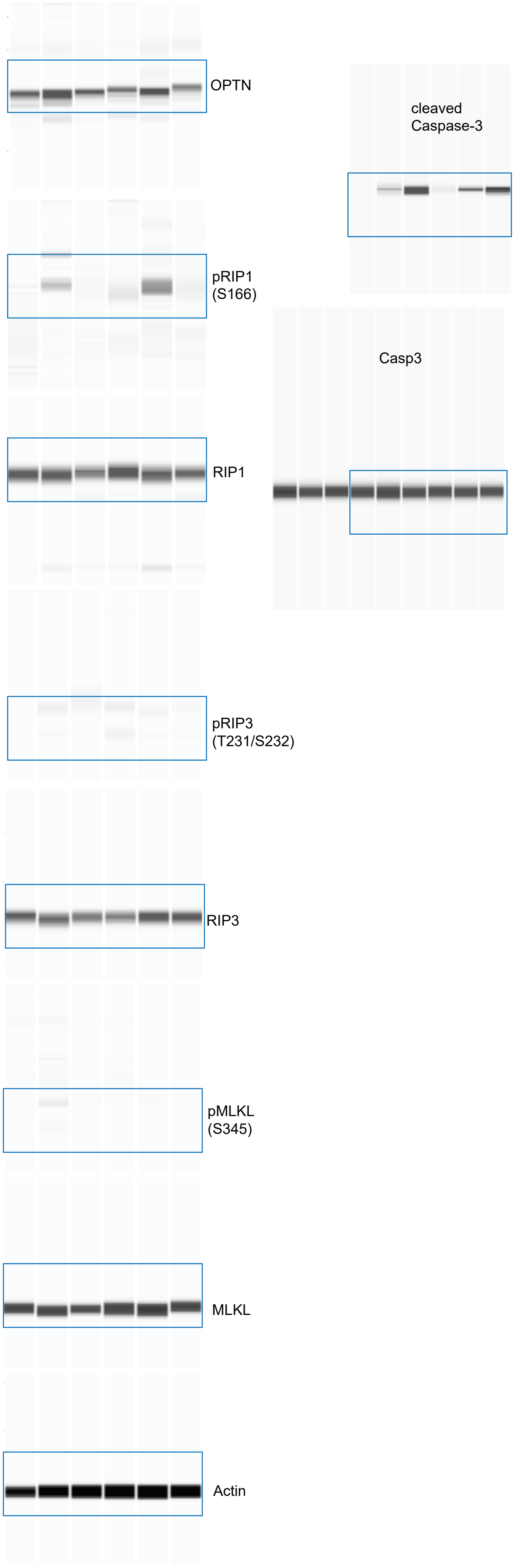

Suppl Figure 3B

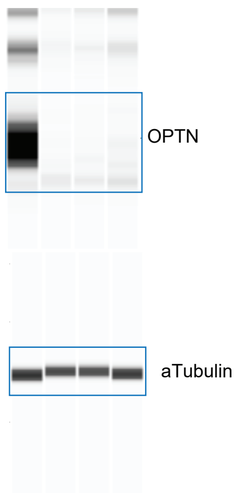

Suppl Figure 3E

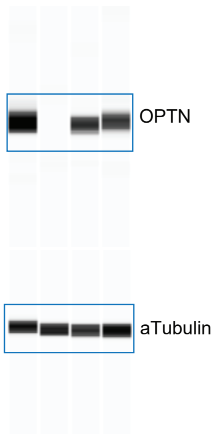

Suppl Figure 3F

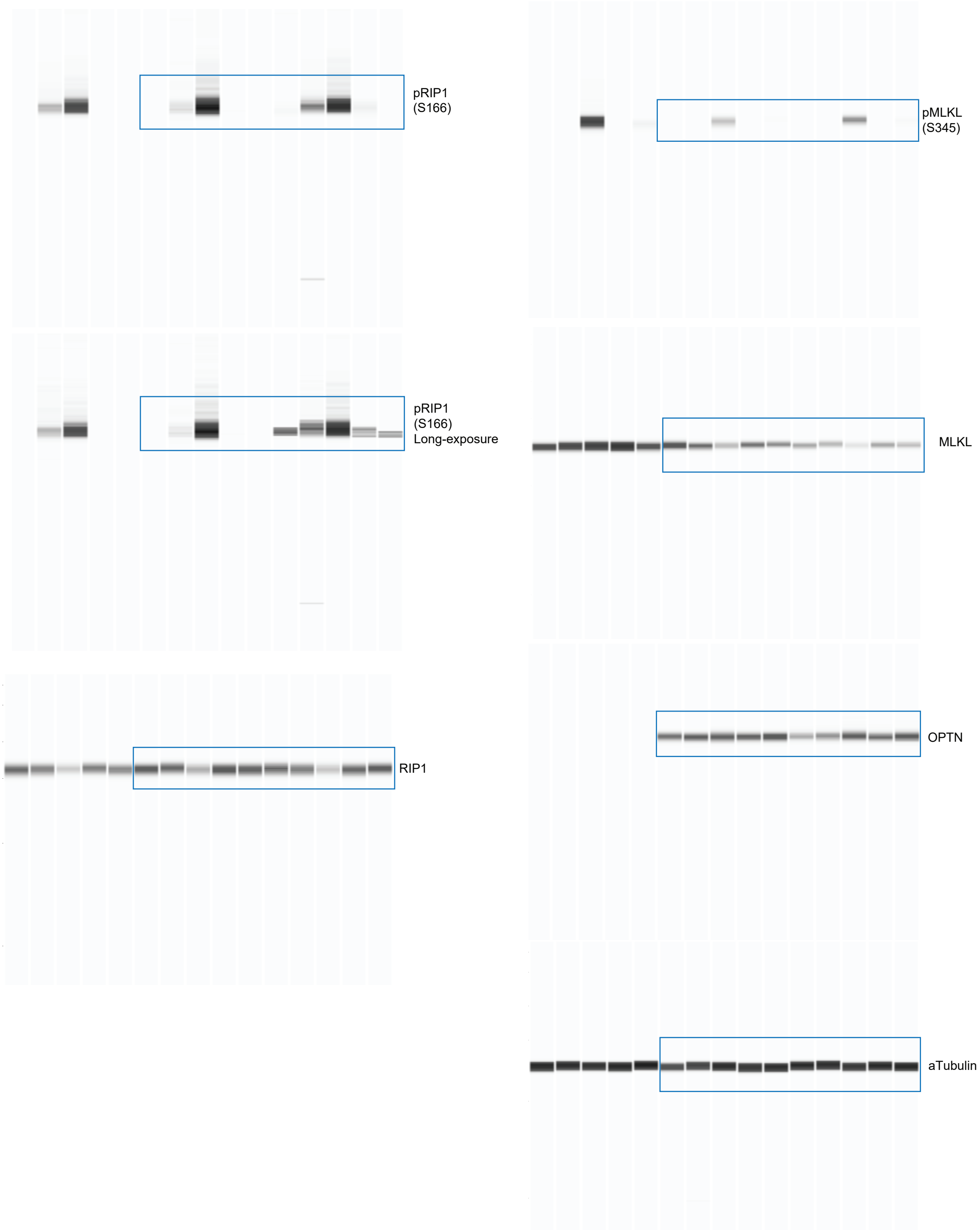

Suppl Figure 4A

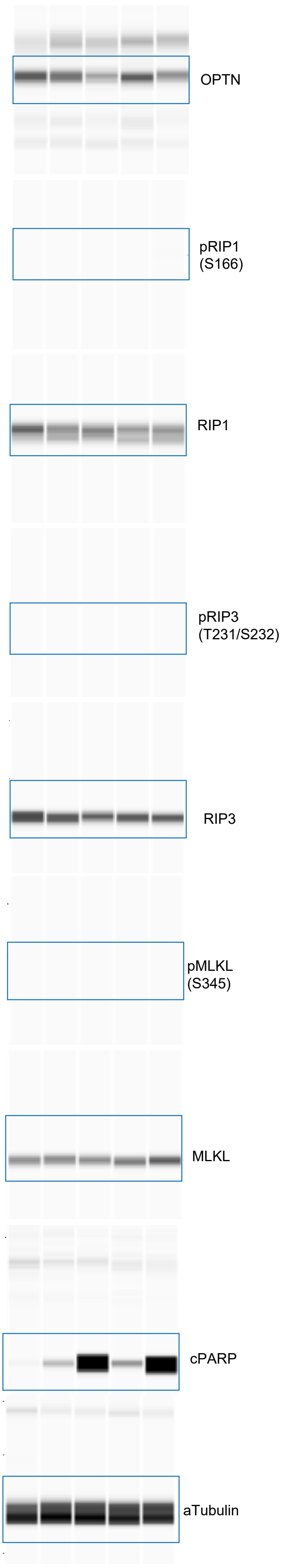

Suppl Figure 4B

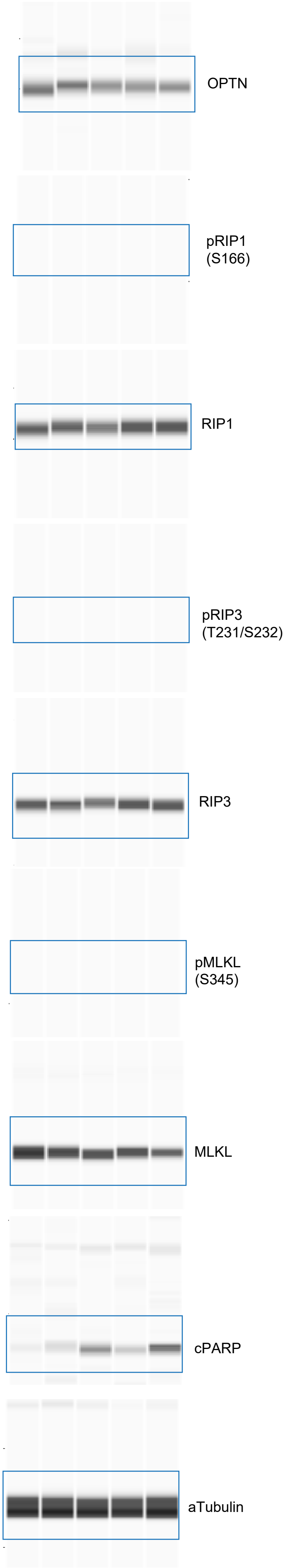

Suppl Figure 4C

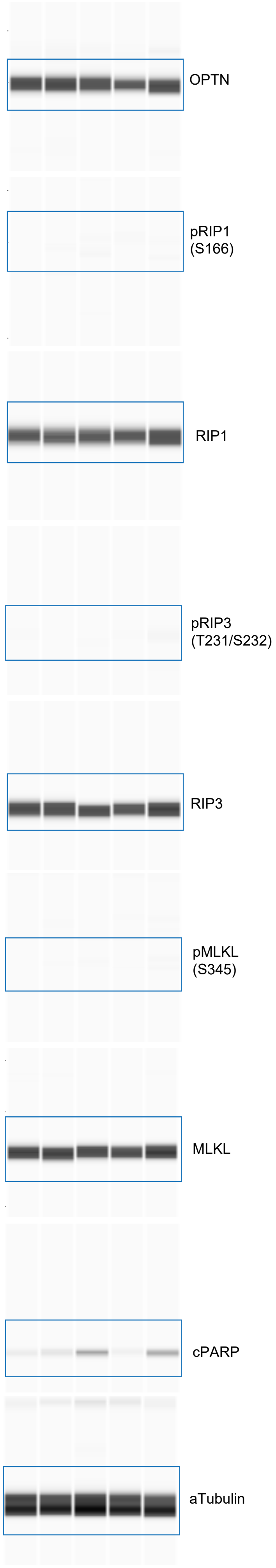

Suppl Figure 5D

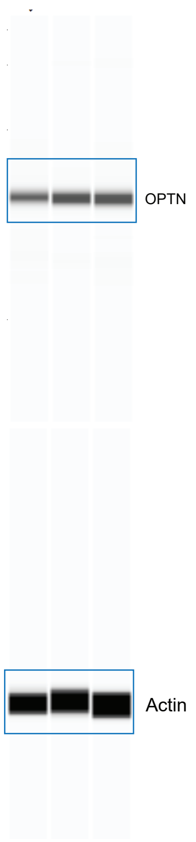

Suppl Figure 5E

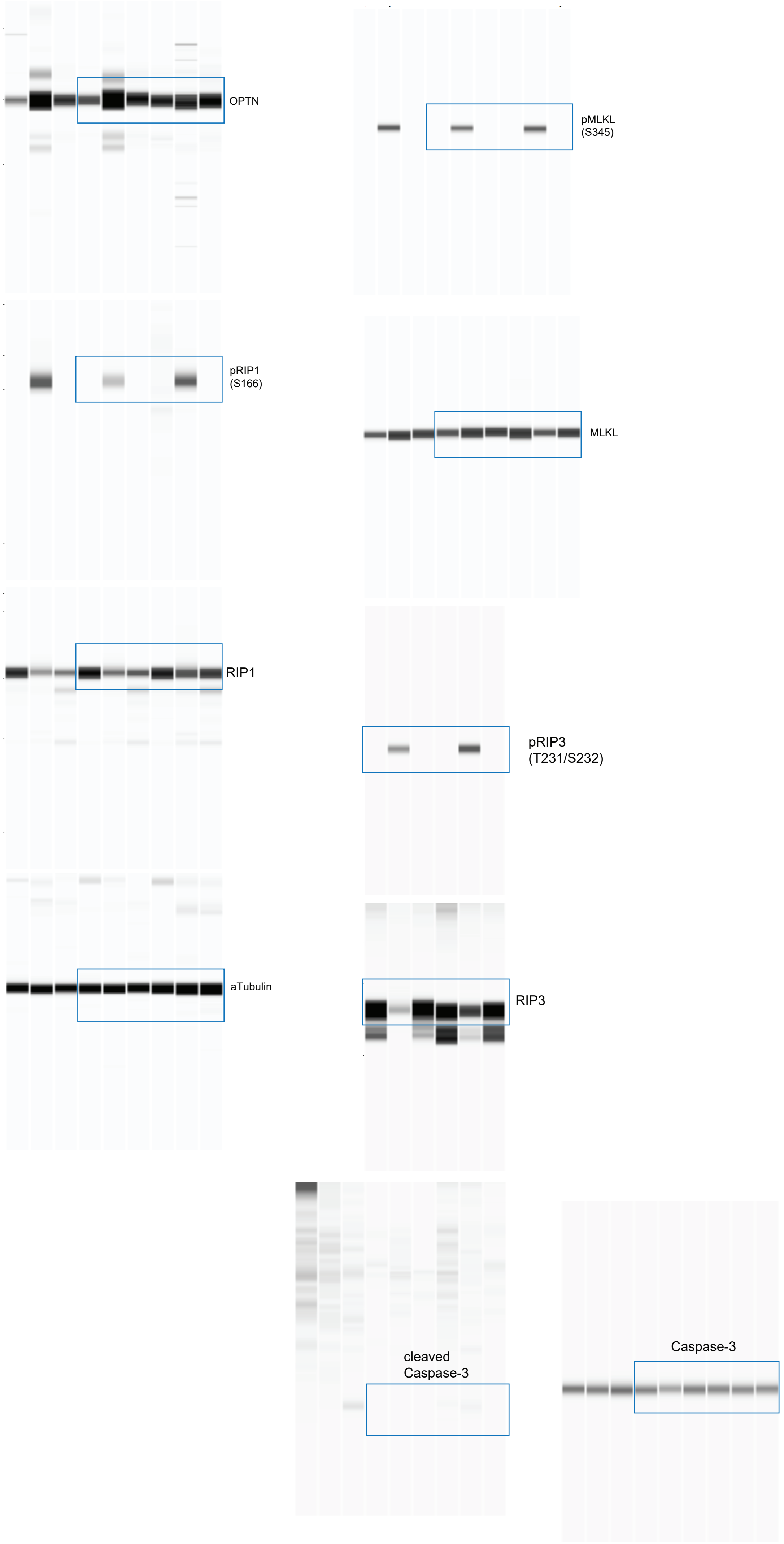

Supplement: Supplementary file 1 — Supplemental Material [file 41418_2024_1390_MOESM1_ESM.pdf]
